# Supplementary material for: Neoadjuvant Camrelizumab Plus Platinum-Based Chemotherapy vs Chemotherapy Alone for Chinese Patients With Resectable Stage IIIA or IIIB (T3N2) Non–Small Cell Lung Cancer: The TD-FOREKNOW Randomized Clinical Trial
Source: JAMA Oncol. 2023 Aug 3;9(10):1348–55. doi: 10.1001/jamaoncol.2023.2751 (PMC10401395; doi:10.1001/jamaoncol.2023.2751)
Supplement: Supplement 1. — Trial Protocol [file jamaoncol-e232751-s001.pdf]

Supplement

The Supplement contains the following items:

1. Trial protocol
2. Amendment history of protocol
3. Statistical analysis plan

An Exploratory Study of Neoadjuvant Camrelizumab in Combination with Nab-Paclitaxel Plus Platinum for Resectable Non-Small Cell Lung Cancer

Protocol No.	XBSX-L1-002
Version No.	V6.0
Version Date	14-OCT-2020
Investigator	Professor Tao Jiang
Sponsor	Department of Thoracic Surgery, The Second Affiliated Hospital of Air Force Medical University

Table of Contents

Signature Page of the Protocol 3

Synopsis 4

Study Flow Chart 10

List of Abbreviations 14

1 Study Background 17

1.1 Epidemiology and Treatment of Lung Cancer 17
1.2 Research Progress of Neoadjuvant Immunotherapy in NSCLC 18
1.3 Research Progress of Immunotherapy Combined with Neoadjuvant Chemotherapy in NSCLC. 19
1.4 Studies of Camrelizumab in NSCLC 20
1.5 Study of Nab-Paclitaxel in NSCLC 25
1.6 Introduction of Camrelizumab 26
1.7 Introduction of nab-paclitaxel 30

2 Study Objectives 33

2.1 Primary Objective 33
2.2 Secondary Objectives 33
2.3 Exploratory Objectives 33

3 Study Design 33

3.1 Overall Design 33
3.2 Sample Size Estimation 34
3.3 Randomization Method 35

4 Selection and Withdrawal of Subjects 35

4.1 Inclusion/Exclusion Criteria 35
4.2 Exclusion Criteria 36
4.3 Withdrawal Criteria 37
4.4 Removal Criteria 38
4.5 Discontinuation Criteria 38

5 Study Drug 38

5.1 Name: Camrelizumab 38
5.2 Name: Nab-paclitaxel 39
5.3 Name: Cisplatin/carboplatin/nedaplatin for injection 39
5.4 Preservation and Management of Drugs 39
5.5 Drug Preparation 40
5.6 Management, Dispensing and Recovery of Drugs 40

6 Dose Regimen 40

6.1 Randomization and Dose 40

6.2 Dose Interruption and Modification 41
6.3 Concomitant Medications and Treatments 43

7 Follow-up 45

8 Clinical Data Collection 46

8.1 Baseline Examinations 46
8.2 On-treatment Examinations 46
8.3 Preoperative Examinations 46
8.4 End of Study Treatment/Withdrawal from the Study 46
8.5 Follow-up 46
8.6 Unscheduled Visits 47

9 Efficacy Evaluation Variables 47

9.1 Primary Efficacy Variable 47
9.2 Secondary Efficacy Variables 47
9.3 Exploratory Efficacy Variable 48

10 Safety Parameters 48

10.1 Adverse Event Observation 48
10.2 Peri- and post-operative complications 50
10.3 Serious Adverse Event 50
10.4 Immune-mediated Adverse Events 51
10.5 Potential Drug-induced Liver Injury 52
10.6 Observation and Management of Adverse Events 52
10.7 Follow-up and Reporting of Adverse Events 54

11 Data Management 56

12 Statistical Analysis 56

12.1 Statistical Hypothesis 56
12.2 Analysis Sets 56
12.3 General Statistical Considerations 57
12.4 Analysis of Primary Efficacy Endpoints 57
12.5 Analysis of Secondary Efficacy Endpoints 57
12.6 Safety Analyses 58

13 Protection of Subject's Rights and Interests 59

14 References 60

Signature Page of the Protocol

I, as a participating doctor/statistician, have read the protocol of this study.

I have fully discussed the objectives of this study and the contents of this protocol with the study director.

I agree to conduct the study in accordance with this protocol, protocol requirements, ethics principles, and under the guidance of Good Clinical Practice (GCP).

I agree that the contents of this protocol will be kept confidential, will not be disclosed to third parties and will only be used for the conduct of this study.

I understand that I will be notified in writing if this study is terminated prematurely or suspended at any time for whatever reason. Similarly, if I decide to withdraw from the conduct of this study, I will immediately notify the leading unit and the Principal Investigators of the study in writing.

Study Unit: Department of Thoracic Surgery, The Second Affiliated Hospital of Air Force Medical University

Signature:________________

Date: ________________

Synopsis

**Study Title**	An Exploratory Study of Neoadjuvant Camrelizumab in Combination with Nab-Paclitaxel Plus Platinum for Resectable Non-Small Cell Lung Cancer
**Version No.**	V6.0
**Version Date**	14 October 2020
**Sponsor**	Tao Jiang
**Subjects**	Patients with resectable stage IIIA-IIIB (IIIB T3N2 only) non-small cell lung cancer (NSCLC)
**Study Objectives**	**Primary objective**<br>To evaluate the pathological complete response (pCR) rate of neoadjuvant camrelizumab in combination with chemotherapy (nab-paclitaxel plus cisplatin/carboplatin/nedaplatin) in resectable NSCLC.
	**Secondary objectives**<br>To evaluate the major pathological response (MPR) rate, objective response rate (ORR), event-free survival (EFS), and safety of neoadjuvant camrelizumab in combination with chemotherapy (nab-paclitaxel plus cisplatin/carboplatin/nedaplatin) in resectable NSCLC.
	**Exploratory Objectives**<br>To assess the feasibility of surgery and rate of peri- and post-operative complications (within 30 days of surgery) in subjects treated with camrelizumab plus chemotherapy compared to those treated with chemotherapy alone.
**Study Design**	A randomised, controlled, open-label, multicenter, phase 2 trial
**Number of Patients Planned to be Enrolled**	A total of 94 subjects will be enrolled and randomized into the treatment group and the control group at a ratio of 1:1, 47 subjects in the treatment group and 47 subjects in the control group.
**Clinical Study Institution**	Department of Thoracic Surgery, The Second Affiliated Hospital of Air Force Medical University
**Patient Screening Criteria**	**Inclusion Criteria:**<br>Patients must meet all of the following inclusion criteria to be eligible for the study:<br>1. 18 to 70 years of age, male or female;<br>2. ECOG PS score 0-1;

3. Expected survival of not less than 12 weeks;
4. Patients with pathologically (histologically or cytologically) confirmed NSCLC (according to the WHO 2015 classification);
5. Patients with resectable stage IIIA-IIIB (IIIB T3N2 only) NSCLC, according to the eighth edition of the clinical TNM staging of tumors;
6. Patients with measurable disease (according to Response Evaluation Criteria in Solid Tumors version 1.1 [RECIST 1.1], the long axis of tumor lesion on CT scan is ≥ 10 mm, or the short axis of lymph node lesion on CT scan is ≥ 15 mm);
7. Patients with initial diagnosis of non-small cell lung before enrollment, without previous radiotherapy, chemotherapy, surgery or targeted therapy;
8. Patients must have adequate cardiopulmonary function for the intended pulmonary resection procedure;
9. The major organ functions are normal, i.e., the following criteria are met:
   (1) Hematology results should meet the following criteria (no blood transfusion, no use of hematopoietic factors, and no drug correction within 14 days):
      a. ANC ≥ 1.5×10^9/L；
      b. PLT ≥ 100×10^9/L；
      c. HB ≥ 90 g/L；
   (2) Blood biochemistry results should meet the following criteria:
      a. TBIL ≤ 1.5×ULN；
      b. ALT and AST ≤ 2.5 × ULN (≤ 5 × ULN if the abnormal liver function is caused by liver metastases);
      c. Serum creatinine (sCr) ≤ 1.5 × ULN and endogenous creatinine clearance ≥ 50 mL/min (Cockcroft-Gault formula);
   (3) Coagulation function must meet: INR ≤ 1.5 × ULN and APTT ≤ 1.5 × ULN;
10. Female subjects of childbearing potential must have a negative serum pregnancy test within 3 days prior to starting study treatment and are willing to use a medically acceptable and highly effective contraceptive method (e.g., intrauterine devices, contraceptives, or condoms) during the study and for 3 months after the last dose of study drug; male subjects with female partners of childbearing potential should be surgically sterile or agree to use effective contraceptive methods during the study and for 3 months after the last dose of study drug.
11. Patients who voluntarily participate in this study, sign informed consent form (ICF), have good compliance, and cooperate with the follow-up.

Exclusion Criteria:

Patients will be ineligible for enrollment into the study if any of the following criteria is met:

1. Patients with metastases to the central nervous system;
2. Patients with a history of any active autoimmune disease or autoimmune disease (including, but not limited to: interstitial pneumonia, uveitis, enteritis, hepatitis, hypophysitis, vasculitis, myocarditis, nephritis, hyperthyroidism, or hypothyroidism [can be allowed after hormone replacement therapy]); patients with vitiligo or childhood asthma which has completely resolved without any intervention indicated in adulthood are allowed to be included; patients requiring medical intervention with bronchodilators are not allowed to be included;
3. Patients with congenital or acquired immune deficiencies, such as human immunodeficiency virus (HIV) infection, active hepatitis B (HBV DNA ≥ 500 IU/ml), hepatitis C (hepatitis C antibody positive and HCV-RNA above the lower limit of detection), or co-infection with hepatitis B and hepatitis C;
4. Patients who have been treated with immunosuppressive drugs, excluding nasal spray and inhaled corticosteroids or physiologic doses of systemic steroids (i.e., no more than 10 mg/day prednisone or its equivalent), within 14 days prior to the first dose of study drug;
5. Patients who have received live attenuated vaccine within 4 weeks prior to the first dose or plan to receive such vaccine during the study;
6. Patients with other malignancies in the past 3 years;
7. Evidence of previous or current pulmonary fibrosis, interstitial pneumonia, pneumoconiosis, radiological pneumonia, drug-induced pneumonia, severe impairment of lung function, etc.;
8. Patients with uncontrolled hypertension (systolic blood pressure ≥ 140 mmHg or diastolic blood pressure ≥ 90 mmHg despite optimal drug therapy);
9. Patients with grade II or higher myocardial ischemia or myocardial infarction, or poorly controlled arrhythmia (including QTc interval ≥ 450 ms in males and ≥ 470 ms in females). Patients with class III-IV cardiac insufficiency by NYHA criteria, or echocardiography suggesting of left ventricular ejection fraction (LVEF) < 50%, or patients with myocardial infarction, New York Heart Association class II or higher cardiac failure, uncontrolled angina pectoris, uncontrolled severe ventricular arrhythmia, clinically significant pericardial disease, or ECG suggesting of acute ischemia or active conduction system abnormalities within 6 months before enrollment;
10. Patients with concurrent severe infection (e.g., requiring IV antibiotics, antifungal or antiviral drugs) within 4 weeks prior to the first dose, or fever of unknown origin > 38.5 °C at screening/prior to the first dose;
11. Patients with a known history of allogeneic organ transplantation or allogeneic hematopoietic stem cell transplantation;

	12. Pregnant or lactating women; or women of childbearing potential who are unwilling or unable to take effective contraceptive measures;<br>13. Known allergy, hypersensitivity, or intolerance to SHR-1210, nab-paclitaxel, or its excipients;<br>14. Patients who are participating in other clinical studies or whose first dose day is less than 4 weeks (or 5 half-lives of the study drug) from the end of the previous clinical study (last dose);<br>15. Patients with a known history of psychotropic drug abuse, alcoholism, or drug addiction;<br>16. Patients with any condition that, in the opinion of the investigator, may jeopardize the patient or render the patient unable to meet or perform the requirements of the study.<br>**Withdrawal Criteria:**<br>1. The subject may withdraw consent and request withdrawal;<br>2. Radiographic evidence of disease progression;<br>3. After study drug is down-titrated to the lowest dose, the subject still can not tolerate it and needs to reduce the dose;<br>4. Other conditions in which withdrawal from the study is deemed necessary by the investigator.<br>5. Lost to follow-up;<br>6. Death of subject.
**Treatment group**	Camrelizumab in combination with nab-paclitaxel and cisplatin/carboplatin/nedaplatin<br>• Camrelizumab: a fixed dose of 200 mg will be administered by intravenous drip over 30 minutes (the overall infusion time, including flush time, is not less than 30 minutes and not more than 60 minutes) on Day 1 of each cycle, every 3 weeks as a cycle.<br>• Nab-paclitaxel: 130 mg/m^2 by intravenous drip over 30 minutes on Day 1 and 8 of each cycle, every 3 weeks as a cycle.<br>• Cisplatin/carboplatin/nedaplatin:<br>Cisplatin: 75 mg/m^2 by intravenous drip on Day 1 of each cycle, every 3 weeks as a cycle.<br>Or carboplatin: AUC5 by intravenous drip on Day 1 of each cycle, every 3 weeks as a cycle.<br>Or nedaplatin: 100 mg/m^2 by intravenous drip on Day 1 of each cycle, every 3 weeks as a cycle.

	Use camrelizumab in combination with nab-paclitaxel plus platinum as neoadjuvant treatment for 3 cycles, off for 4 to 6 weeks, and then followed by surgery, for resectable NSCLC.
**Control Group**	Nab-paclitaxel and cisplatin/carboplatin/nedaplatin<br>• Nab-paclitaxel: 130 mg/m^2 by intravenous drip over 30 minutes on Day 1 and 8 of each cycle, every 3 weeks as a cycle.<br>• Cisplatin/carboplatin/nedaplatin:<br>Cisplatin: 75 mg/m^2 by intravenous drip on Day 1 of each cycle, every 3 weeks as a cycle.<br>Or carboplatin: AUC5 by intravenous drip on Day 1 of each cycle, every 3 weeks as a cycle.<br>Or nedaplatin: 100 mg/m^2 by intravenous drip on Day 1 of each cycle, every 3 weeks as a cycle.<br>Use neoadjuvant treatment of nab-paclitaxel plus platinum for 3 cycles, off for 4 to 6 weeks, and then followed by surgery, for the resectable NSCLC.
**Endpoints Assessment**	**I. Primary Endpoint**<br>Pathological complete response (pCR) rate: defined as the proportion of patients with no residual tumor cells in the surgically resected tumor specimen and all sampled regional lymph nodes after neoadjuvant treatment.<br>**II. Secondary Endpoints**<br>Major pathological response rate (MPR) rate: defined as the proportion of patients with ≤ 10% of residual tumor cells in the surgically resected tumor specimen and sampled regional lymph nodes after neoadjuvant treatment.<br>Objective response rate (ORR): defined as the proportion of patients whose tumor size shrinks to predefined values prior to definitive surgery, including cases of radiographic complete response (CR) and partial response (PR). Objective tumor response will be assessed using RECIST 1.1. Subjects must have measurable tumor lesions at baseline, and the response evaluation criteria are classified as CR, PR, stable disease (SD), and progressive disease (PD) according to RECIST 1.1.<br>Event-free survival (EFS): defined as the time from the randomization to the first occurrence of disease progression or recurrence, or death (due to any cause).<br>Safety: AEs will be graded into 1-5 grades according to NCI Common Acute and Subacute Toxicity Grading Criteria (NCI-CTCAE 5.0).<br>**III. Exploratory Endpoints**<br>To assess the feasibility of surgery and rate of peri- and post-operative complications (within 30 days of surgery) in subjects treated with camrelizumab plus chemotherapy

	compared to those treated with chemotherapy alone.
**Statistical methods**	Demographic and baseline characteristics will be summarized descriptively.<br><br>Primary efficacy analysis will be primarily performed in FAS. The 95% confidence interval of pCR will be estimated by using Clopper-Pearson method. A two-sided 95% confidence interval for odds ratio of pCR between the treatment groups will also be computed. Fisher's exact test will be used to test the difference of pCR between treatment groups. A p-value (two-sided) less than 0.05 will be regarded as statistically significant.<br><br>For binary secondary efficacy endpoints, including MPR and ORR, the number and proportion of subjects in each treatment group will be presented. The 95% confidence interval will be estimated by using Clopper-Pearson method. A two-sided 95% confidence interval for odds ratio between the treatment groups will also be computed. For time-to-event secondary efficacy endpoints, the Kaplan-Meier method will be used to estimate the distribution of EFS in each treatment group, the 95% confidence intervals of median EFS will be calculated by using Brookmeyer and Crowley method, and the 95% confidence intervals of EFS rates by complementary log-log method. The corresponding Kaplan-Meier plots will also be presented. In addition, the unstratified Cox proportional hazards regression model will be used to estimate the hazard ratio and the associated 95% confidence interval.<br><br>Safety endpoints and surgical related endpoints will be summarized descriptively.

Study Flow Chart

Item \ Treatment cycle	Screening		Treatment			7 days prior to the scheduled surgery date	4-6 weeks after the surgery	Follow-up (after end of treatment)	
	d-14~ d-1	d-7~ d-1	C1	C2	C3			Safety follow-up [19]	Survival follow-up [20]
Sign the ICF	x								
Demographic data	x								
Medical and treatment history	x								
Inclusion criteria reconciliation		x							
ECOG PS score [1]		x	x	x	x	x	x	x	
Vital signs [2]		x	x	x	x	x	x	x	
Physical examination [3]		x	x	x	x	x	x	x	
Virological tests[4]	x								
Hematology [5]		x		x	x	x	x	x	
Urinalysis [6]		x		x	x	x	x	x	
Blood chemistry [7]		x		x	x	x	x	x	
Stool routine [8]		x		x	x	x	x	x	
Coagulation [9]		x		x	x	x	x	x	
Thyroid function test [10]		x		x	x	x	x	x	
12-lead ECG [11]		x		x	x	x	x	x	
Echocardiography [12]		x							
Pregnancy test [13]		x				x	x	x	
Tumor imaging [14]	x					x		x	x
Pulmonary function assessment [15]	x					x			
Pathological assessment [16]	x						x		
AEs [17]	x	x	x	x	x	x	x	x	x
Concomitant medications/treatments	x	x	x	x	x	x	x	x	x

Treatment cycle / Item	Screening		Treatment			7 days prior to the scheduled surgery date	4-6 weeks after the surgery	Follow-up (after end of treatment)	
	d-14~ d-1	d-7~ d-1	C1	C2	C3			Safety follow-up [19]	Survival follow-up [20]
[18]									
Cardiac enzymes [21]		x		x	x	x	x	x	
Genetic testing [22]							x		
Study treatment [23]			x	x	x				

Note: in addition to the tests and time points listed in the table, the investigator may add visits and other tests as needed, and the test results should be filled in the corresponding part of the case report form (e.g., Unscheduled Visit Tests). The planned time window for this study is ± 3 days, unless otherwise noted.

[1] ECOG PS score: it will be performed within 7 days prior to the first dose, prior to dosing on Day 1 of each treatment cycle, 7 days before surgical resection, 3-6 weeks after surgery, at the end of treatment/withdrawal from the study, and at the first visit during the safety follow-up period.

[2] Vital signs: including pulse, respiratory rate, body temperature, and blood pressure; it will be performed within 7 days prior to the first dose, prior to dosing on Day 1 of each treatment cycle, 7 days before surgical resection, 3-6 weeks after surgery, at the end of treatment/withdrawal from the study, and at the first visit during the safety follow-up period.

[3] Physical examination: a complete physical examination (general condition, head and face, skin, lymph nodes, eyes, ears, nose, throat, mouth, respiratory system, cardiovascular system, abdomen, genitourinary system, muscles and bones, nervous system, mental status, etc.) will be performed prior to dosing on Day 1 of each treatment cycle and at the end of treatment/withdrawal from the study; a physical examination will be performed within 7 days prior to the first dose, prior to dosing on Day 1 of each treatment cycle, 7 days before surgical resection, 3-6 weeks after surgery, at the end of treatment/withdrawal from the study, and at the first visit during the safety follow-up period.

[4] Virological tests: including HBsAg, HBsAb, HBeAg, HBeAb, HBcAb, HBV DNA (qualitative test, quantitative test if positive), HCV-Ab (quantitative HCV-RNA test if positive), and HIV-Ab.

[5] Hematology: including red blood cell count (RBC), hemoglobin (Hb), platelet count (PLT), white blood cell count (WBC), neutrophil count (ANC), and lymphocyte count; it will be performed within 7 days prior to the first dose, prior to dosing on Day 1 of the second and third treatment cycle, 7 days before surgical resection, 3-6 weeks after surgery, at the end of treatment/withdrawal from the study, and at the first visit during the safety follow-up period.

[6] Urinalysis: including white blood cells, red blood cells, and urine protein. It will be performed within 7 days prior to the first dose, prior to dosing on Day 1 of the second and third treatment cycles, 7 days before surgical resection, 3-6 weeks after surgery, at the end of treatment/withdrawal from the study, and at the first visit during the safety follow-up period.

[7] Blood chemistry: including alanine aminotransferase (ALT), aspartate aminotransferase (AST), glutamyl transpeptidase (γ-GT), total bilirubin (TBIL), direct bilirubin (DBIL), alkaline

phosphatase (AKP), blood urea nitrogen (BUN), total protein (TP), albumin (ALB), creatinine (Cr), blood glucose (GLU), K^+, Na^+, Ca^{2+}, Mg^{2+}, and Cl^-; it will be performed within 7 days prior to the first dose, prior to dosing on Day 1 of the second and third treatment cycles, 7 days before surgical resection, 3-6 weeks after surgery, at the end of treatment/withdrawal from the study, and at the first visit during the safety follow-up period.

[8] Stool routine: it will be performed within 7 days before the first dose, prior to dosing on Day 1 of the second and third treatment cycles, 7 days before surgical resection, 3-6 weeks after surgery, at the end of treatment/withdrawal from the study, and at the first visit during the safety follow-up period. (If fecal occult blood is positive, reexamination is required; if reexamination of fecal occult blood is still positive, gastrointestinal endoscopy should be performed). Thereafter, it will be performed based on the investigator's clinical judgment.

[9] Coagulation: including activated partial thromboplastin time (APTT), prothrombin time (PT), thrombin time (TT), fibrinogen (FIB), and international normalized ratio (INR); it will be performed within 7 days prior to the first dose, prior to dosing on Day 1 of the second and third treatment cycles, 7 days before surgical resection, 3-6 weeks after surgery, at the end of treatment/withdrawal from the study, and at the first visit during the safety follow-up period.

[10] Thyroid function: including serum thyroid stimulating hormone (TSH), free triiodothyronine (FT3), and free thyroxine (FT4); if FT3 and FT4 are not available, T3 and T4 may be used; it will be performed within 7 days prior to the first dose, prior to dosing on Day 1 of the second and third treatment cycles, 7 days before surgical resection, 3-6 weeks after surgery, at the end of treatment/withdrawal from the study, and at the first visit during the safety follow-up period.

[11] 12-lead ECG: attention should be paid to QT, QTc, and P-R intervals. It will be performed within 7 days prior to the first dose, prior to dosing on Day 1 of the second and third treatment cycles, 7 days before surgical resection, 3-6 weeks after surgery, at the end of treatment/withdrawal from the study, and at the first visit during the safety follow-up period.

[12] Echocardiography: it will be performed within 7 days prior to randomization and at the end-of-treatment visit, and may be performed if there are clinically significant ECG abnormalities during the study.

[13] Pregnancy test: for women of childbearing potential, serum pregnancy test will be used. It will be performed within 7 days prior to the first dose, 1 week before surgery, 3-6 weeks after surgery, at the end of treatment/withdrawal from the study, and at the first visit during the safety follow-up period.

[14] Tumor imaging: including chest CT and abdominal ultrasound; PET-CT and cranial MRI (1.5 T plain + enhanced scan, which may be replaced by cranial CT if the patient is contraindicated for MRI) may be performed within 60 days prior to the first dose of study drug; bone scan must be performed within 42 days prior to the first dose and should be performed only if clinically indicated.

- ✓ At screening, tumor assessment may be performed within 4 weeks prior to the first dose of study drug, and tumor imaging result prior to signing of informed consent may be used for tumor assessment at screening as long as it meets the RECIST 1.1 requirements.
- ✓ Complete imaging assessment will be performed within 7 days prior to lung cancer surgery.
- ✓ Subjects who do not have radiographic progression during the safety follow-up visit and survival follow-up visit should still undergo radiographic assessments at the same frequency until disease progression or initiation of other anti-tumor therapy.
- ✓ In addition to radiologically confirmed PD, subjects who discontinue the treatment for other reasons should also undergo imaging examination at the frequency specified in the

protocol as far as possible until documented PD, initiation of new anti-tumor therapy, or death.

[15] Pulmonary function assessment: normal pulmonary function or mild to moderate abnormal pulmonary function (VC% > 60%, FEV1 > 1.2 L, FEV1% > 40%, DLco > 40%), and can tolerate lung cancer resection; the assessment should be performed once within 14 days prior to the first dose and 7 days before lung cancer resection.

[16] Pathological assessment: pathological diagnosis and assessment will be performed within 90 days before the first dose and after lung cancer resection.

[17] AEs: recorded from the time of signing the ICF to 90 days after the last dose. AEs should be followed until resolution, resolved to baseline or ≤ Grade 1, stabilized, or reasonably explained (e.g., lost to follow-up, death).

[18] Concomitant medications/treatments: concomitant medications/treatments will be recorded from 14 days prior to the first dose of study medication to the end of the safety visit, and only concomitant medications/treatments for AEs related to the use of study drug will be recorded after the end of study drug treatment or withdrawal from the study.

[19] Safety follow-up: it will be performed every 30 days (± 3 days) from the last study treatment until 90 (± 7 days) after the last study treatment. The first safety follow-up visit should be conducted at the study site to complete the protocol-specified examinations and assessments; the subsequent safety follow-up visit will be conducted by telephone and only collect survival information, concomitant medications/treatments, and AEs.

[20] Survival follow-up: after the end of the safety follow-up period, the subjects will enter the survival follow-up period until the subject death, lost to follow-up, withdrawal of informed consent, or study termination by the sponsor. During this period, the subjects will be followed every 3 months in the first and second years, every 6 months from the third year to the fifth year, and annually thereafter to collect survival and subsequent treatment information.

[21] Myocardial zymogram: including creatine kinase isoenzyme (CK-MB), α-hydroxybutyrate dehydrogenase, creatine kinase, lactate dehydrogenase, etc. It will be performed within 7 days prior to randomization, prior to dosing in the second and third treatment cycles, before and after surgery, and at the safety follow-up visit; it will be supplemented if symptoms such as precordial pain, palpitations, and ECG abnormalities occur during the study, and at the end of treatment.

[22] Genetic testing: if the subject has previously undergone EGFR mutation testing, ALK fusion gene testing, etc., these testings will not be repeated; if the subject has not undergone relevant testings, the site should perform relevant testing according to the patient's wishes. It is recommended that the EGFR mutation should be tested using ARMS or Super ARMS, and the ALK fusion gene should be tested using Ventana immunohistochemistry, FISH or RT-PCR.

[23] Study treatment: treatment group: camrelizumab + nab-paclitaxel + cisplatin/carboplatin/nedaplatin; control group: nab-paclitaxel + cisplatin/carboplatin/nedaplatin; detailed description is provided in the study treatment section.

List of Abbreviations

Abbreviation	Definition
AE	Adverse event
AKP	Alkaline phosphatase
ALT	Alanine aminotransferase
ANC	Absolute neutrophil count
APTT	Activated partial thromboplastin Time
AST	Aspartate aminotransferase
BIL	Bilirubin
BUN	Blood urea nitrogen
Cr	Creatinine
CCr	Creatinine clearance
CR	Complete response
CRF	Case report form
CT	Computed tomography
CTCAE	Common Terminology Criteria for Adverse Events
CFDA	China Food and Drug Administration
DLT	Dose limiting toxicity
ECOG	Eastern Cooperative Oncology Group
ECG	Electro cardio gram
EORTC	The European Organization for Research and Treatment for Cancer
Fbg	Fibrinogen

Abbreviation	Definition
GCP	Good Clinical Practice
FAS	Full analysis set
Glu	Glucose
Hb	Hemoglobin
HR	Hazard ratio
INR	International normalized ratio
MRI	Magnetic resonance imaging
MPR	major pathologic response
MTD	Maximum tolerated dose
NTAX	Nanoparticle albumin-bound-paclitaxel
NSCLC	Non-small cell lung cancer
OB	Occult blood
ORR	Objective response rate
PD	Progressive disease
pCR	Progressive disease
PI	Principal investigator
PLT	Platelets
PRO	Protein
PK	Pharmacokinetics
PD	Pharmacodynamics
PPS	Per protocol set
PR	Partial response

Abbreviation	Definition
PS	Performance status
PT	Prothrombin time
RBC	Red blood cell
RECIST	Response Evaluation Criteria in Solid Tumor
RR	Response rate
γ-GT	γ-glutamyltransferase
SAE	Serious adverse event
SAS	Safety analysis set
SCr	Serum creatinine
SD	Stable disease
TT	Thrombin time
ULN	Upper limit of normal
WBC	White blood cell

1 Study Background

1.1 Epidemiology and Treatment of Lung Cancer

The etiology of lung cancer is still not completely clear, and a large number of data indicate that long-term heavy smoking is closely related to the occurrence of lung cancer. Previous studies have shown that long-term heavy smokers are 10 to 20 times more likely to develop lung cancer than nonsmokers. The younger the age at which they start smoking, the higher the chance of developing lung cancer. In addition, smoking not only directly affects one's own health, but also has a negative impact on the health of the surrounding population, resulting in a significant increase in the prevalence of lung cancer in passive smokers. The incidence of lung cancer is higher in urban residents than in rural residents, which may be related to urban air pollution and the presence of carcinogens in smoke and dust [1].

Lung cancer has become the leading cause of cancer death in both men and women worldwide. According to the statistics in the 2012 Chinese Cancer Registration Annual Report, in China, lung cancer ranks the first among cancers, both in terms of incidence and mortality. According to the 2012 Cancer Registration Annual Report, the incidence of lung cancer is 54/100,000, and the mortality is 48/100,000, which is a serious threat to people's health. Among lung cancers, patients with non-small cell lung cancer (NSCLC) account for about 80% of all lung cancer patients. Internationally, the treatment of NSCLC is generally surgical treatment, chemoradiotherapy and immunotherapy. Surgical treatment is still the first choice, which can significantly improve the quality of life of patients and prolong their postoperative survival. For patients with stage II and IIIA NSCLC, the surgical resection rate is less than 30%, the 5-year survival rate after surgery is very low, and the recurrence rate is high within a short time after surgery. For preoperative neoadjuvant chemotherapy for stage IB, II and IIIA NSCLC, relevant studies have demonstrated that chemotherapeutic drugs, targeted therapy and immunotherapy can significantly reduce tumor stage, inhibit tumor cell recurrence and metastasis, and significantly enlarge the space between tumor cells and trachea, blood vessels, surrounding tissues and mediastinal lymph nodes, so as to reduce the difficulty of surgical operation, shorten the operation time, reduce intraoperative blood loss, increase the complete resection rate of tumors, and reduce postoperative complications. Therefore, preoperative neoadjuvant chemotherapy

combined with surgery is of great significance for patients in stages IB, II and IIIA, and provides a reliable and sound theoretical basis for clinical work. The theoretical advantages are: ① reducing the tumor stage and decreasing the tumor volume, thereby increasing the surgical resection rate; ② controlling and treating micrometastases in the body, thereby reducing the recurrence rate; ③ chemotherapeutic drugs can reach the tumor tissue in sufficient amounts through the undamaged blood supply system; ④ evaluating the in vivo sensitivity of chemotherapeutic drugs to guide postoperative treatment [2] .

1.2 Research Progress of Neoadjuvant Immunotherapy in NSCLC

At present, immunotherapy has become a neoadjuvant treatment for tumors. A study [3] presented at the 2018 AACR Meeting introduced the application of nivolumab as neoadjuvant treatment in resectable NSCLC. The study results showed that in 20 of 21 patients, the tumor was completely resected. Of these 20 patients with completely resected tumors, 9 had a significant pathological response (defined as less than 10% viable cancer cells in the tumor specimen). It's concluded that nivolumab as neoadjuvant immunotherapy for NSCLC had few adverse effects and did not delay the timing of surgery, and that 45% of tumors would have a significant pathological response. Study LCMC3 [4] was an open-label, single-arm Phase 2 study of atezolizumab as neoadjuvant and adjuvant therapy in untreated patients with stage IB/II or IIIA resectable NSCLC. Patients with EGFR mutations or ALK fusions were excluded from the study. Of the 45 patients who received atezolizumab and underwent surgical resection, 3 (7%) patients achieved pCR and 10 (22%) patients achieved mPR (major pathological response), but no patients in the TC0/IC0 subgroup achieved pCR or mPR. Study NEOSTAR [5] presented at the 2018 ESMO Congress was a single-center, open-label Phase 2 study that included patients with resectable stage I-IIIA NSCLC, randomized into Group A and Group B in a ratio of 1:1. Group A received neoadjuvant monotherapy of nivolumab, and Group B received neoadjuvant treatment of ipilimumab in combination with nivolumab. Of the 26 evaluable patients who received surgical treatment, the proportion of patients achieving pathological complete response (pCR) in the postoperative specimen was 19%, with 14% and 25% in the two groups, respectively; the proportion of patients achieving mPR was 11%, with 14% and 8% in the two groups, respectively. The median proportion of viable cells in the surgical specimen was 65% and 27.5% in the two groups, respectively. Safety assessments

showed that neoadjuvant treatment was well tolerated, with the majority of treatment-related adverse events (TRAEs) of Grade 1-2. The incidences of Grade 1-2 TRAEs were 59%, 27.1%, and 72.9% in the overall population and two groups, respectively. This finding extends the experience of neoadjuvant treatment for NSCLC and introduces a combination treatment strategy based on existing neoadjuvant single-agent immunotherapy.

1.3 Research Progress of Immunotherapy Combined with Neoadjuvant Chemotherapy in NSCLC

The main mechanism by which paclitaxel inhibited tumor growth is to arrest the cell cycle at G2/M phase by stabilizing microtubules, interfering with microtubule motility and then activating the spindle checkpoint, thereby inhibiting cell division and inducing apoptosis. The efficiency of specific stabilization of the tumor cell microtubule system by paclitaxel is partially counteracted by β-tubulin, so the antitumor effect of paclitaxel has been confirmed to act on the tumor cell microtubule system. It was found that paclitaxel also affected the function and structure of mitochondria, but the relationship between this microtubule dysfunction, the change of mitochondrial membrane permeability and the release of cytochrome C was not well understood. According to Kutuk et al, paclitaxel could induce competitive replacement of Bim proteins of the BH3 domain protein family by Bmf and Puma proteins, thereby activating Bax and Bak proteins to induce apoptosis. Paclitaxel could also induce Caspase-8-dependent apoptosis by affecting the microtubule-associated death effector domain. Park et al have shown that paclitaxel could promote chromosome terminal lysis through telomere depletion, thereby prolonging arrest at G2/M phase and multinucleation. Multinucleated telomere dysfunction could be another important cause of apoptosis. In addition, cell autophagy is also directly involved in the inhibitory effect of paclitaxel on tumor cells. The results showed that paclitaxel could effectively increase the number of fluorescently labeled particles of autophagic vacuoles of v-Ha-ras-transfected NIH3T3 fibroblasts by fluorescent dye monodansylcadaverine (MDC), but neither phagocytosis inhibitor nor apoptosis inhibitor completely blocked the cell death induced by paclitaxel, suggesting that both phagocytosis and apoptosis may be involved

in the destruction of tumor cells by paclitaxel.

At the 2018 WCLC Assembly, the immunotherapy in combination with chemotherapy of paclitaxel plus platinum showed superior results. Study NADIM [6] was a Phase 2, single-arm, open-label, multicenter study enrolling patients with locally advanced resectable stage IIIA N2 NSCLC. The patients received chemotherapy in combination with adjuvant treatment of nivolumab for 1 year. The neoadjuvant treatment regimen consisted of 3 cycles of nivolumab 360 mg IV Q3W in combination with paclitaxel 200 mg/m^2 and carboplatin AUC6 IV Q3W chemotherapy. Tumor assessment was performed after completion of neoadjuvant treatment and prior to surgery. Surgery was performed at Week 3 or 4 after completion of 3 cycles of neoadjuvant treatment. Adjuvant treatment regimen included nivolumab 240 mg IV Q2W for 4 months and nivolumab 480 mg IV Q4W for 8 months, with a total of 1 year after surgery. A total of 46 patients were included in the study, 20 of whom completed surgery. Chemotherapy in combination with immunotherapy was well tolerated and no patient was extended for care. No patient was withdrawn from the study prior to surgery due to disease progression or toxicity. A total of 20 patients received the surgery and all tumors were resectable. In the assessment of overall clinical response rate, 5% of patients achieved CR and 65% of patients achieved pCR. In the assessment of response rate in postoperative cases, 13 patients (65.0%; 95% CI 40.8%-84.6%) achieved pathological complete response, and 3 (15.0%) patients achieved major pathological response, defined as < 10% of viable tumor cells in the resected specimen. Based on the patients who achieved pathological complete response and major PR, the overall ORR was 80.0% (95% CI 56.3%-94.3%) and the CR rate was 60%. Chemotherapy in combination with immunotherapy was assessed in the neoadjuvant treatment period, which demonstrated superior anti-tumor efficacy in patients with locally advanced, potentially resectable NSCLC, and achieved a pCR rate beyond expectation.

1.4 Studies of Camrelizumab in NSCLC

1.4.1 Safety Summary of Phase 1 Study of SHR-1210 in the Treatment of Advanced Solid Tumors

After clinical trial approval was obtained in 2016, 3 phase 1 clinical studies of SHR-1210 was conducted in China, all of which were safety and tolerability studies in patients with advanced solid tumors. As of 28 February 2018, a total of 258 patients

with advanced solid tumors who failed standard of care were included in the 3 Phase 1 studies, and their safety is summarized and analyzed as follows:

All 258 subjects (100.0%) experienced at least one AE. AEs with an incidence of ≥ 10% mainly included: skin and subcutaneous tissue disorders: reactive cutaneous capillary hyperplasia (81.8%), pruritus (22.5%), and rash (16.3%); hepatic function abnormal: aspartate aminotransferase increased (22.1%), alanine aminotransferase increased (19.0%), bilirubin conjugated increased (17.8%), and blood bilirubin increased (13.2%); hematologic toxicity: anaemia (29.5%), white blood cell count decreased (17.1%), and neutrophil count decreased (10.0%); general symptoms: asthenia (38.4%), and pyrexia (22.1%); gastrointestinal AEs: nausea (12.0%), and diarrhoea (11.6%); respiratory, thoracic and mediastinal disorders: cough (21.3%), and upper respiratory tract infection (10.9%); metabolism and nutrition disorders: hypoproteinaemia (22.1%), blood sodium decreased (18.2%), and decreased appetite (12.0%); renal and urinary disorders: proteinuria (22.5%); endocrine disorders: hypothyroidism (20.9%). A total of 98 (38.0%) subjects experienced at least one Grade 3 or higher AE. Grade 3 or higher AEs occurring in ≥ 2% of subjects mainly included anaemia (7.0%), lung infection (6.6%), blood sodium decreased (4.3%), bilirubin conjugated increased (3.9%), neoplasm progression (3.5%), death (3.1%), aspartate aminotransferase increased (2.7%), alanine aminotransferase increased (2.3%), and blood bilirubin increased (2.3%).

In the 258 subjects, 256 (99.2%) subjects had at least one drug-related AE. Drug-related AEs with an incidence of ≥ 10% mainly included: skin and subcutaneous tissue disorders: reactive cutaneous capillary hyperplasia (81.8%), pruritus (22.1%), and rash (16.3%); general symptoms: asthenia (37.6%), and pyrexia (20.9%); hepatic function abnormal: aspartate aminotransferase increased (21.7%), alanine aminotransferase increased (18.6%), bilirubin conjugated increased (16.7%), and blood bilirubin increased (12.0%); hematologic toxicity: anaemia (27.5%), and white blood cell count decreased (14.7%); gastrointestinal disorders: diarrhoea (11.2%), and nausea (10.0%); respiratory, thoracic and mediastinal disorders: cough (19.0%), and upper respiratory tract infection (10.1%); metabolism and nutrition disorders: hypoproteinaemia (19.4%), and blood sodium decreased (14.3%); renal and urinary disorders: proteinuria (22.1%);

endocrine disorders: hypothyroidism (19.8%). Eighty-two (31.8%) subjects experienced at least one Grade 3 or higher drug-related AE. Grade 3 or higher drug-related AEs occurring in ≥ 2% of subjects mainly included anaemia (6.2%), lung infection (5.8%), bilirubin conjugated increased (3.9%), blood sodium decreased (3.5%), death (3.1%), aspartate aminotransferase increased (2.7%), alanine aminotransferase increased (2.3%), and blood bilirubin increased (2.3%).

In the 258 subjects, Grade 3 or higher AEs occurring in ≥ 2% of subjects mainly included anaemia (7.0%), lung infection (6.6%), blood sodium decreased (4.3%), bilirubin conjugated increased (3.9%), neoplasm progression (3.5%), death (3.1%), aspartate aminotransferase increased (2.7%), alanine aminotransferase increased (2.3%), and blood bilirubin increased (2.3%).

Overall, the AEs of SHR-1210 in patients with advanced solid tumors were similar to those of the marketed equivalent drugs, except for reactive cutaneous capillary hyperplasia. Reactive cutaneous capillary hyperplasia mostly occurred within 1-2 months after the start of study treatment, the early cases occurred a few days after administration, and the later cases occurred about 5 months after administration, mainly in the trunk or extremities. The vast majority (99.5%) were CTCAE Grade 1-2, and only 1 subject was reported as Grade 3, which was assessed as Grade 3 AE due to hospitalization as a result of surgical resection No patient discontinued due to this AE. A small proportion of subjects with bleeding symptom were treated with local symptomatic treatment and the symptom generally resolved gradually after discontinuation of SHR-1210 treatment. Overall, SHR-1210 was safe and well tolerated.

1.4.2 Preliminary Results of SHR-1210 Monotherapy Study in NSCLC

1) SHR-1210-II-201-NSCLC: this study is an ongoing open-label, single-arm, multicenter Phase 2 study to assess the efficacy and safety of camrelizumab as second-line therapy in subjects with advanced or metastatic NSCLC who have previously been treated with at least one platinum-based doublet chemotherapy regimen. In the study, the subjects were divided into 4 groups according to PD-L1 expression: a) PD-L1 expression < 1% group; b) PD-L1 expression ≥ 1% and < 25% group; c) PD-L1

expression ≥ 25% and < 50% group; and d) PD-L1 expression ≥ 50% group. The study was conducted in 2 periods. In Period 1, subjects were assigned to the corresponding group based on PD-L1 expression level, and Period 2 would only be entered if there was at least 1 responder (with objective response) in Period 1. After PD-L1 testing, the subjects were assigned to the corresponding group to receive camrelizumab. As of the data cut-off date of 25 August 2018, a total of 146 subjects were enrolled, and the preliminary safety and exposure results of the study were as follows: 146 subjects with NSCLC received 1 to 16 cycles of camrelizumab monotherapy. Fifty-four (37.0%) subjects withdrew from study treatment due to disease progression. Seven (4.8%) subjects discontinued study treatment due to TEAEs. One hundred and forty-two subjects (97.3%) reported AEs during the study. The most frequently reported TEAEs were RCCEP (69.2%), anaemia (21.2%), and cough (16.4%). SAEs were reported in 43 subjects (29.5%). SAEs occurring in > 2 subjects were disease progression (5.5%), haemoptysis, pneumonitis (3.4% each), lung infection, RCCEP, and respiratory failure (2.1% each). Fifteen subjects (10.3%) had at least one uncoded AE; 3 subjects (2.1%) had an uncoded SAE.

2) SHR-1210-APTN-II-202-NSCLC: this is an ongoing Phase 2 study to evaluate the safety and efficacy of camrelizumab in combination with apatinib in subjects with non-squamous NSCLC who have metastasized or progressed after prior multimodal therapy. As of the data cutoff date of 25 August 2018, a total of 85 subjects were enrolled. The preliminary safety and exposure results of the study were as follows: 76 subjects with NSCLC received 1 to 16 cycles of camrelizumab in combination with apatinib. Nine subjects (10.6%) discontinued the study treatment due to disease progression. Nine subjects discontinued the study treatment due to TEAEs. Sixty-two subjects (81.6%) had treatment-emergent AEs. The most frequently reported TEAEs were proteinuria (35.5%), hypertension (34.2%), AST increased, PPE syndrome (26.3% each), pyrexia (25.0%), decreased appetite (22.4%), ALT increased and rash (21.1% each). Twenty-three subjects (30.3%) had SAEs. SAEs occurring in ≥ 2 subjects were disease progression (5.3%), lung infection (3.9%), pyrexia, and autoimmune hepatitis (2.6%

each). Seventeen subjects (22.4%) had at least one uncoded adverse event; one subject had an uncoded SAE.

3) SHR-1210-III-303-NSCLC: this is an ongoing open-label, randomized, multicenter Phase 3 study to assess the efficacy and safety of camrelizumab in combination with pemetrexed plus carboplatin as first-line treatment in subjects with advanced or metastatic non-squamous NSCLC. As of the data cutoff date of 25 August 2018, a total of 418 subjects were enrolled, including 208 subjects in the treatment group and 210 subjects in the control group. The preliminary safety and exposure results of the study were as follows: in the treatment group (camrelizumab in combination with pemetrexed + carboplatin), 205 subjects with NSCLC received 1-20 cycles of treatment with camrelizumab. In the control group (pemetrexed plus carboplatin), 207 subjects with NSCLC received 1 to 21 cycles of pemetrexed in combination with carboplatin or pemetrexed monotherapy. After the randomization period, 54 subjects (26.1%) in the control group crossed over to receive camrelizumab. Fifteen subjects (7.3%) in the treatment group and 12 subjects (5.8%) in the control group discontinued the study treatment due to TEAEs. Two hundred and four subjects (99.5%) in the treatment group and 201 subjects (97.1%) in the control group experienced AEs during the treatment; 45 subjects (83.3%) in the control group who crossed over to receive camrelizumab experienced AEs during the treatment. In the treatment group, the most frequently reported TEAEs were neutrophil count decreased (66.3%), white blood cell count decreased (64.4%), anaemia (59.5%), and RCCEP (66.8%). In the control group, the most frequently reported TEAEs were neutrophil count decreased (60.9%), white blood cell count decreased (59.4%), and anaemia (53.6%). The most frequently reported TEAEs in subjects in the control group who crossed over to receive camrelizumab were RCCEP (29.6%) and anaemia (20.4%). SAEs were reported in 64 subjects (31.2%) in the treatment group and 40 subjects (19.3%) in the control group. SAEs were reported in 9 subjects (16.7%) in the control group who crossed over to receive camrelizumab. In the treatment group, SAEs with an incidence of > 1% included platelet count decreased (7.3%), bone marrow failure (3.9%), lung infection (2.9%), anaemia (2.4%), ALT increased, respiratory failure (2.0% each), disease progression, hepatic function

abnormal, upper respiratory tract infection, interstitial lung disease, pneumonitis, neutrophil count decreased, and white blood cell count decreased (1.5% each). In the control group, SAEs with an incidence of > 1% included death (2.4%), platelet count decreased, lung infection (1.9% each), neutrophil count decreased, white blood cell count decreased, dyspnoea, and pleural effusion (1.4% each). In subjects in the control group who crossed over to receive camrelizumab, SAEs with an incidence of > 1% included lung infection, disease progression, haemoptysis (3.7% each), pneumonitis, respiratory failure, acute coronary syndrome, gastrointestinal haemorrhage, and tumour thrombosis (1.9% each). Forty-four subjects (21.5%) in the treatment group and 29 subjects (14.0%) in the control group reported uncoded AEs; 5 subjects (9.3%) in the control group who crossed over to receive camrelizumab reported uncoded AEs. Two subjects (1.0%) in the treatment group and 1 subject (0.5%) in the control group reported uncoded SAEs; no subject in the control group who crossed over to receive camrelizumab reported an uncoded SAE.

Clinically significant laboratory results were reported as AEs, without other measures of special interest. Vital signs were stable with no extreme values.

1.5 Study of Nab-Paclitaxel in NSCLC

Nab-paclitaxel in combination with carboplatin is recommended as first-line treatment for NSCLC based on a randomized, open-label, international multicenter study CA031 [8]. Of 1052 patients with treatment-naïve stage IIIB or IV NSCLC, 521 patients were randomized to receive nab-PC 100 mg/m^2 (Day 1, 8, and 15) in combination with carboplatin AUC6 for 21 days as a cycle, and 531 patients were randomized to receive sb-PC 200 mg/m^2 in combination with carboplatin AUC6 for 21 days as a cycle. The ORR (33% vs 25%, P = 0.005), median PFS (6.3 vs 5.8 months, P = 0.21), and median OS (12.1 vs 11.2 months, P = 0.27) were higher in the nab-PC group than in the sb-PC group. In patients with squamous NSCLC, the ORR increased by 71% (41% vs 24%, P < 0.001) and the median OS increased 1.2 months (10.7 vs 9.5 months, P = 0.284) in the nab-PC group than in the sb-PC group. In patients with non-squamous NSCLC, the efficacy was comparable (ORR: 26% vs 25%, P = 0.808) in the two groups. In patients

older than 70 years of age, the ORR increased by 40% (34% vs 24%, P = 0.196), the median PFS increased 1.2 months (8 vs 6.8 months, P = 0.134), and the median OS increased 9.5 months (19.9 vs 10.4 months, P = 0.009) in the nab-PC group than in the sb-PC group. The incidences of Grade 3 or higher neuritis peripheral, neutropenia, arthralgia, and myalgia were significantly less in the nab-PC group than in the sb-PC group, while the incidence of anaemia and thrombocytopenia were higher. In summary, nab-PC regimen is safe and effective as the first-line treatment for advanced NSCLC, with more significant efficacy for squamous NSCLC, and high efficiency, low toxicity and good tolerability in elderly patients with lung cancer.

In view of the superior results of the above-mentioned anti-PD-1 antibody monotherapy or in combination with paclitaxel plus platinum as the neoadjuvant treatment of NSCLC, it is hoped to use camrelizumab (SHR-1210) combined with nab-paclitaxel and cisplatin/carboplatin/nedaplatin premedication to increase the tumor resection rate and further benefit the patients with stage IIIA-IIIB (stage IIIB T3N2 only) NSCLC.

1.6 Introduction of Camrelizumab

1.6.1 Pharmacological Type and Mechanism of Action

Programmed death-1 (PD-1) is a protein receptor expressed on the surface of T cells, which participates in the process of cell apoptosis. PD-1 belongs to the CD28 family and shares 23% amino acid identity with cytotoxic T lymphocyte antigen 4 (CTLA-4), but its expression is different from that of CTLA-4, mainly expressed on activated T cells, B cells and myeloid cells. PD-1 has two ligands, i.e. PD-L1 and PD-L2. PD-L1 is mainly expressed on T cells, B cells, macrophages and dendritic cells (DCs), and can be up-regulated on activated cells. Whereas the expression of PD-L2 is relatively limited, it is mainly expressed on antigen-presenting cells, such as activated macrophages and dendritic cells. The humanized anti-PD1 monoclonal antibody is able to specifically bind to PD-1 and block the interaction of PD-1 with its ligand, allowing T cells to restore immune responses against tumors.

1.6.2 Pharmacodynamic studies

The results of the binding affinity study for antibody SHR-1210 to human, monkey and murine antigens (Table 1) showed that the affinities of SHR-1210 to human and monkey PD1 antigens were 6.9 nM and 4.1 nM, respectively, which were fairly close,

while there was no measurable binding to murine PD-1 antigen. The affinity of SHR-1210 for the antigen (human PD-1) was 3.0 nM and its activity was comparable to that of the control antibodies Nivolumab and Pembrolizumab (Table 2).

Table 1 Reactive Affinity of Antibody SHR-1210 Binding to Human, Monkey and Murine PD-1 Antigens

Stationary Phase	Mobile Phase	Affinity (nM)
SHR-1210	Human PD-1 antigen	6.9
SHR-1210	Murine PD-1 antigen	Very weak signal, no measurable binding
Monkey PD-1 antigen (-hFc)	SHR-1210	4.1

Table 2 Blockade of PD-1/PD-L1 Binding by Antibody SHR-1210

Antibody	Antigen	Affinity (nM)
SHR-1210	Human PD-1 antigen	3.0
Nivolumab	Human PD-1 antigen	4.0
MK3475	Human PD-1 antigen	3.2

The results of antibody SHR-1210 blocking PD-1/PD-L1 binding study (Figure 1 and Figure 2) showed that SHR-1210 showed comparable *in vitro* blocking activity on PD-1/PD-L1 binding with Nivolumab and Pembrolizumab. The blocking activity IC50 of antibodies SHR-1210, Nivolumab and Pembrolizumab were 0.70 nM/0.79 nM and 0.79 nM/0.77 nM, respectively.

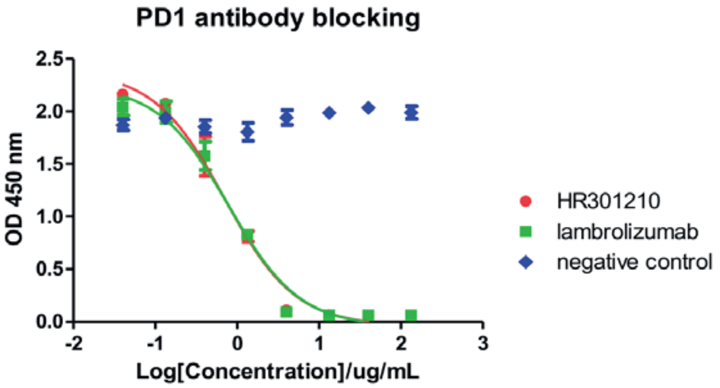

	HR301210	lambrolizumab
IC50	0.7007	0.7864

Figure 1 Blockade of PD-1/PD-L1 Binding by SHR-1210 and Pembrolizumab

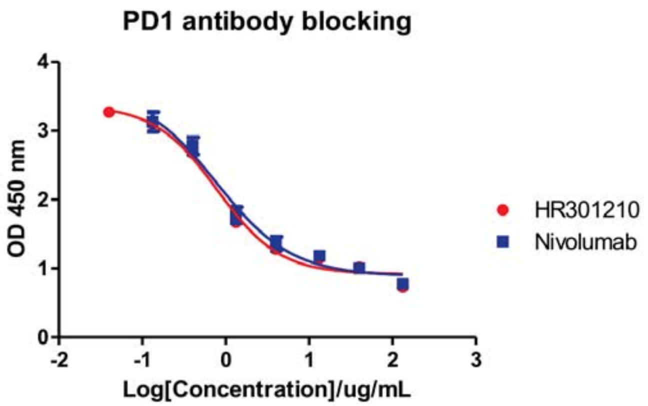

	HR301210	Nivolumab
IC50	0.7913	0.7745

Figure 2 Blockade of PD-1/PD-L1 Binding by SHR-1210 and Nivolumab

1.6.3 Toxicology Studies

In the preclinical acute toxicity study in cynomolgus monkeys, 8 cynomolgus monkeys (half males and half females) were randomly divided into 2 groups. Animals in Group 2 were administered SHR-1210 via intravenous injection at doses of 200, 400 and 800 mg/kg every other day in a dose escalation fashion. There were no SHR-1210-related changes in clinical symptoms, body weights, food consumption and hemagglutination. Decreased lymphocytes were observed in both sexes at doses ≥ 200 mg/kg; increased serum globulin and decreased albumin were observed in both sexes at doses ≥ 400 mg/kg. These changes were not considered to be harmful due to their small magnitude. The maximum tolerated dose (MTD) of SHR-1210 was ≥ 800 mg/kg.

In the completed preclinical long-term toxicity study in cynomolgus monkeys, SHR-1210 was well tolerated by intravenous injection at 20, 50 and 100 mg/kg once weekly for 4 weeks (5 doses in total) in both sexes. There were no SHR-1210-related clinical symptoms, including injection site irritation, or changes in body weight, food consumption, body temperature, ECG, blood pressure, heart rate and respiratory parameters; there were also no changes in B- and T-lymphocyte typing, cytokines, immunoglobulin and complement parameters, and no SHR-1210-related changes in organ weights, macroscopic lesions and histopathological.

1.6.4 PK Studies

PK parameters following a single intravenous infusion of SHR-1210 to cynomolgus monkeys are presented in Table 3.

Table 3 PK Parameters Following a Single Intravenous Infusion of SHR-1210 at Different Doses in Cynomolgus Monkeys

Dose (mg/kg)	Sex	T1/2 (hr)	Tmax (hr)	Cmax (μg/ml)	AUClast (hr×μg/ml)	Vz (ml/kg)	Cl (ml/hr/kg)	MRTlast (hr)
1	Females	76.06±32.93	0.83±0.29	31.16±11.25	1716.12±453	54.09±14.85	0.57±0.17	80.95±18.58
	Males	91.72±25.26	0.83±0.29	35.96±13.09	2359.7±684.07	55.15±20.51	0.37±0.06	102.23±38.56
	Overall	83.89±27.62	0.83±0.26	33.56±11.23	2037.91±627.32	54.62±16.02	0.47±0.15	91.59±29.47
2	Females	92.95±22.60	0.83±0.29	81.09±12.66	6896.79±1673.36	40.75±12.66	0.44±0.11	120.92±49.96
	Males	113.54±8.26	1.67±0.58	71.65±10.85	6380.24±2062.85	47.05±27.05	0.47±0.12	127.10±59.24
	Overall	103.25±18.94	1.25±0.61	76.37±11.74	6638.51±1703.60	43.91±19.21	0.46±0.11	124.01±49.13
3	Females	169.70±38.96	2.17±1.76	217.46±20.22	31357.28±9338.28	41.24±24.76	0.33±0.1	179.68±73.6
	Males	128.94±35.93	0.67±0.29	251.88±6.49	26779.98±7205.43	30.9±30.2	0.31±0.05	113.25±44.39
	Overall	149.32±40.28	1.42±1.39	234.67±23.15	29068.63±7869.83	36.07±25.34	0.32±0.07	146.46±65.42

1.6.5 Clinical Study Progress of SHR-1210

Since 2015, Hengrui has conducted 4 phase I clinical studies at multiple sites in Australia and China to preliminarily validate the safety, tolerability and efficacy of SHR-1210 in patients with advanced solid tumors who have failed existing standard of care.

As of the end of 2016, a total of 140 subjects with solid tumors were enrolled at home and abroad, including a total of 116 subjects enrolled in China and 24 subjects in Australia. The tolerability observation results showed that different doses (1 mg/kg, 3 mg/kg, 6 mg/kg, 10 mg/kg) and fixed doses (60 mg, 200 mg and 600 mg) of SHR-1210 were well tolerated, and no dose-limiting toxicity was observed in any dose group during the tolerability observation period, i.e. the MTD exceeded 10 mg/kg or 600 mg. Adverse reactions judged by the investigator to be possibly related to the study drug (SHR-1210) mainly included: reversible capillary hyperplasia, rash, pruritus, fatigue, asthenia, pyrexia, anemia, nausea, vomiting, headache, dizziness, diarrhea, serum transaminase increased, serum bilirubin increased, QT interval prolongation, hypothyroidism or hyperthyroidism and hypophysitis, etc. The majority of adverse reactions were grade 1 to 2 and could be effectively controlled with or without medical intervention.

At the 2018 ASCO meeting, professor Zhou Caicun, Chest Hospital of Tongji University, issued the results of a phase IB clinical study of apatinib in combination with SHR-1210 (PD-1) in patients with advanced NSCLC. The study enrolled 27 patients with advanced NSCLC who failed second-line or above chemotherapy and were randomized to SHR-1210, 200 mg, q2w in combination with apatinib 250 mg qd

or SHR-1210, 200 mg, q2w, in combination with apatinib 375 mg. The results showed the ORR = 41.2% and DCR = 94.1% in the apatinib 250 mg group. The median PFS was 24 weeks. Relative to the 375 mg dose group, one patient in the apatinib 250 mg group interrupted the SHR-1210 administration, and there were two adverse reactions: one bronchopleural fistula and one anorexia. In conclusion, apatinib 250 mg in combination with SHR-1210 showed good tolerability and anti-tumor efficacy in the treatment of NSCLC patients.

1.7 Introduction of nab-paclitaxel

Paclitaxel (albumin-bound) is a Cremophor EL-free lyophilized formulation with human serum albumin as excipient. It is produced by high-pressure micro-jet emulsification, evaporation and freeze-drying processes, and the suspension with an average particle size of 100 nm to 200 nm can be formed after redispersion with normal saline. Its albumin moiety binds to a specific albumin cell surface receptor (gp60) on the surface of vascular endothelial cells, activating caveolin-1 which is the major component of cell membrane vesicles, leading to receptor-mediated entry of the albumin drug complex into cell membrane caveolae, followed by transmembrane transport, and the drug entry into tumor cells. The uptake and accumulation of nab-paclitaxel in the tumor tissue space is enhanced by the interaction between albumin and SPARC (cysteine-rich acidic secretory protein) proteins. SPARC secreted by tumors, which function similar to albumin receptors, can specifically attract and adhere to albumin. Therefore, SPARC proteins can specifically adsorb cytotoxic drugs that bound to albumin and aggregate them on tumor cells, thereby increasing local drug concentration and enhancing the killing ability to tumors.

As a new formulation of paclitaxel, this product can be infused without a cosolvent and can effectively avoid adverse reactions related to organic solvents. Compared with the conventional formulation of paclitaxel, the infusion time of this product is shortened from 3 hours to 30 minutes, and prophylactic anti-allergic treatment is not required before administration; its clinical application is safer and convenient. At the same time, the improved safety allows patients to tolerate higher doses of paclitaxel, and its good targeting and sustained-release characteristics also enhances the dose-response

relationship, predicting a stronger anti-tumor effect of the product in humans.

1.7.1 Nomenclature

The chemical name is: 5β, 20-epoxy-1, 2α, 4, 7β, 10β, 13α-hexahydroxytaxan-11-en-9-one-4,10-diacetate-2-benzoate-13- (2R, 3S) -N-benzoyl-3-phenylisoserine ester, the molecular formula is $C_{47}H_{51}NO_{14}$, and the molecular weight is 853.91, as shown in Figure 3.

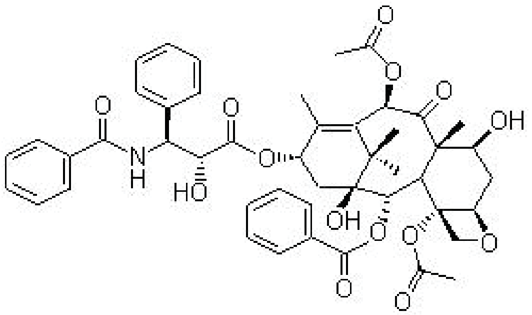

Figure 3 Molecular Structure of Paclitaxel (Albumin-bound)

1.7.2 PK Studies [4]

Absorption

PK parameters were studied by the innovator company at doses ranging from 80 to 375 mg/m^2 when completed within 30 or 180 minutes. The results showed that with the infusion of the drug, the blood concentration of paclitaxel for injection (albumin-bound) showed a biphasic decrease, in which the rapid decrease represented the distribution of the drug to surrounding tissues, and the slow phase represented the clearance of the drug. The terminal half-life was approximately 27 hours.

At 80 to 375 mg/m^2, AUC was positively correlated with dose; PK parameters were not related to continuous administration. For metastatic breast cancer, a dose of 260 mg/m^2 was administered with a maximum paclitaxel concentration of 18741 ng/mL, which occurred at the end of the injection. The mean clearance was 15 L/hr/m^2 with a mean distribution of 632 L/m^2 in peripheral tissues. Paclitaxel for injection (albumin-bound) at 260 mg/m^2 was administered as a 30-minute infusion, and the clearance and distribution of paclitaxel for injection (albumin-bound) was higher than that of paclitaxel injection when compared to paclitaxel injection at 175 mg/m^2 as a 3-hour infusion. Differences in C_{max} and dose-corrected C_{max} reflected differences in total amount and injection rate. There was no difference in the final half-life between the two.

Distribution

89% to 98% of paclitaxel at concentrations of 0.1 to 50 μg/mL bound to serum proteins in the *in vitro* studies. Cimetidine, ranitidine, dexamethasone, or diphenhydramine did not affect the binding of paclitaxel to protein.

Metabolism

In vitro studies on liver microsomes and liver tissue sections showed that paclitaxel was metabolized primarily to 6α-hydroxypaclitaxel by CYP2C8, and to two minor metabolites, i.e. 3'-p-hydroxypaclitaxel and 6α-, 3'-p-hydroxypaclitaxel via CYP3A4. *In vitro*, the metabolism of paclitaxel to 6α-hydroxypaclitaxel was inhibited by some drugs such as ketoconazole, verapamil, diazepam, quinidine, dexamethasone, cyclosporine, teniposide, etoposide and vincristine. Testosterone, 17α-ethinyl estradiol, retinoic acid and quercetin, a specific inhibitor of CYP2C8, also inhibited the formation of 6α-hydroxypaclitaxel *in vitro*. PK parameters of paclitaxel were also affected in the *in vitro* studies when mixed with CYP2C8 and/or CYP3A4 as substrates, inducers, or inhibitors.

Excretion

Paclitaxel for injection (albumin-bound) at 260 mg/m^2 was injected over 30 minutes and the cumulative mean unchanged drug in urine was 4%, indicating that it is not metabolized by the kidney. Less than 1% was excreted in urine as 3'-p-hydroxypaclitaxel and 6 α-, 3'-p-hydroxypaclitaxel. Approximately 20% was excreted in feces.

1.7.3 Special Populations

Hepatic impairment:

The innovator company assessed the PK parameters of paclitaxel for injection (albumin-bound) over 30 minutes in 15 patients with solid tumors and hepatic impairment. During the course of the study, the dosing was based on the degree of hepatic impairment in patients at the following doses:

Mild (1-fold ULN < bilirubin ≤ 1.25-fold ULN, 1-fold ULN < AST < 10-fold ULN): 260 mg/m^2

Moderate (1.26-fold ULN < bilirubin ≤ 2-fold ULN, 1-fold ULN < AST < 10-fold ULN): 200 mg/m^2

Severe (2.01-fold ULN < bilirubin ≤ 5-fold ULN, 1-fold ULN < AST < 10-fold ULN): 130 mg/m^2

Table 4 PK Parameter AUC_{inf} (hr * ng/mL) in Patients with Hepatic Impairment

	Mild (n = 5)	Moderate (n = 5)	Severe (n = 5)
Dose	260 mg/m^2	200 mg/m^2	130 mg/m^2
Mean±SD	17434±11454	14159±13346	9187±6475
Mean (range)	13755（7618,35262）	7866（5919,37613）	6134（5627,20684）

Renal impairment:

The effect of renal impairment on paclitaxel for injection (albumin-bound) has not been studied.

2 Study Objectives

2.1 Primary Objective

To evaluate the pathological complete response (pCR) rate of neoadjuvant camrelizumab in combination with chemotherapy (nab-paclitaxel plus cisplatin/carboplatin/nedaplatin) in resectable NSCLC.

2.2 Secondary Objectives

To evaluate the major pathological response (MPR) rate, objective response rate (ORR), event-free survival (EFS), and safety of neoadjuvant camrelizumab in combination with chemotherapy (nab-paclitaxel plus cisplatin/carboplatin/nedaplatin) in resectable NSCLC.

2.3 Exploratory Objectives

To assess the feasibility of surgery and rate of peri- and post-operative complications (within 30 days of surgery) in subjects treated with camrelizumab plus chemotherapy compared to those treated with chemotherapy alone.

3 Study Design

3.1 Overall Design

This is a randomized controlled, open-label, multi-center exploratory study to assess the efficacy and safety of neoadjuvant camrelizumab in combination with chemotherapy (nab-paclitaxel plus cisplatin/carboplatin/nedaplatin) in resectable stage IIIA-IIIB (IIIB T3N2 only) NSCLC. The overall design is shown in Figure 4.

Ninety-four subjects with stage IIIA-IIIB (IIIB T3N2 only) NSCLC are planned to be enrolled and randomized in a ratio of 1:1 to receive either camrelizumab in combination with nab-paclitaxel plus cisplatin/carboplatin/nedaplatin or nab-paclitaxel in combination with cisplatin/carboplatin/nedaplatin. Each subject will receive 3 cycles of study treatment followed by surgery 4 to 6 weeks after the discontinuation. Patients will be discontinued from treatment if they experience the following conditions during treatment, such as disease progression, intolerable toxic and side effects of the drug, withdrawal of ICF, etc. Efficacy and safety variables will be observed during the study.

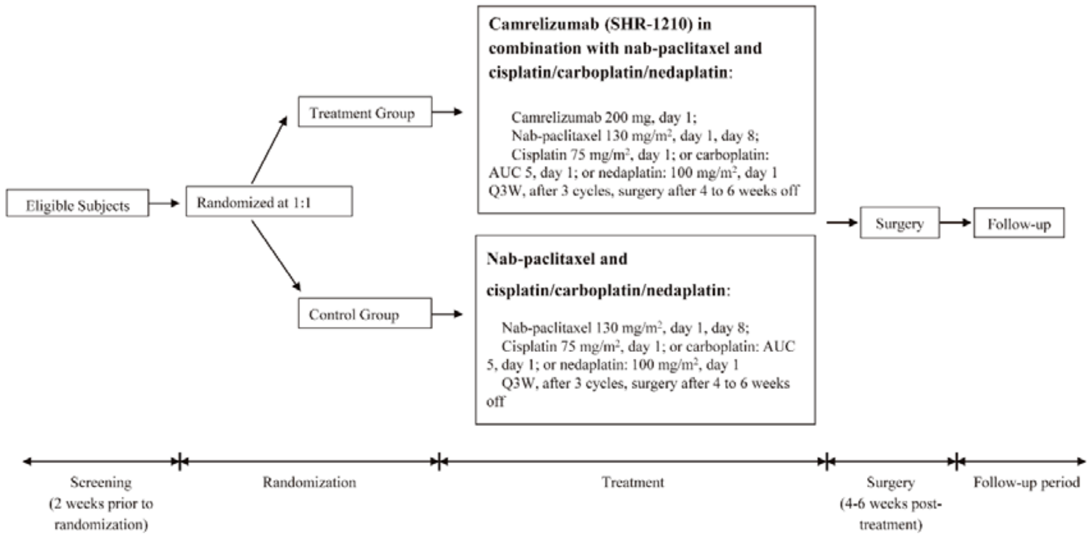

Figure 4. Overall Schematic Diagram of Study Design

3.2 Sample Size Estimation

Sample size will be based on the primary endpoint in the primary analysis. pCR rate in the camrelizumab plus chemotherapy group is assumed to be 50% and 18% in the chemotherapy alone group, which can be translated to an odds ratio of 4.55. Under these assumption, 80 subjects will provide at least 80% power to detect the difference between two groups with a two-sided alpha level of 0.05 in the primary analysis (i.e., in the full analysis set which is a modified ITT population to include all randomized subjects who received at least 1 dose of study treatment). Accounting for a potential 15% dropout after randomization, we plan to enroll 94 patients.

3.3 Randomization Method

The eligible subjects will be randomly assigned to the camrelizumab plus chemotherapy group or the chemotherapy alone group at a ratio of 1:1 by permuted block randomization method. The treatment allocation will be implemented via opaque, sealed envelopes. As this is an open-label study, patients and investigators will not be masked to the treatment allocation.

4 Selection and Withdrawal of Subjects

4.1 Inclusion/Exclusion Criteria

Inclusion Criteria:

1) 18 to 70 years of age, male or female;
2) ECOG PS score 0-1;
3) Expected survival of not less than 12 weeks;
4) Patients with pathologically (histologically or cytologically) confirmed NSCLC (according to the WHO 2015 classification);
5) Patients with resectable stage IIIA-IIIB (IIIB T3N2 only) NSCLC, according to the eighth edition of the clinical TNM staging of tumors;
6) Patients with measurable disease (according to Response Evaluation Criteria in Solid Tumors version 1.1 [RECIST 1.1], the long axis of tumor lesion on CT scan is ≥ 10 mm, or the short axis of lymph node lesion on CT scan is ≥ 15 mm;);
7) Patients with initial diagnosis of NSCLC before enrollment, without previous radiotherapy, chemotherapy, surgery or targeted therapy;
8) Patients must have adequate pulmonary function for the intended pulmonary resection procedure;
9) The major organ functions are normal, i.e., the following criteria are met:

(1) Hematology test should meet the following criteria:

a) $ANC \geq 1.5 \times 10^9/L$；

b) $PLT \geq 100 \times 10^9/L$；

c) $HB \geq 90g/L$；

(2) Results of blood biochemistry tests should comply with the following criteria:

a) $TBIL \leq 1.5 \times ULN$；

b) ALT and AST < 2.5 × ULN, and ALT and AST < 5 × ULN for patients with

liver metastases;

c) BUN and Cr ≤ 1.5 × ULN or endogenous creatinine clearance ≥ 50 mL/min (Cockcroft-Gault formula).

(3) Coagulation function must meet: INR ≤ 1.5 and APTT ≤ 1.5 × ULN;

10) Female subjects of childbearing potential must have a negative serum pregnancy test within 3 days prior to starting study treatment and are willing to use a medically acceptable and highly effective contraceptive method (e.g., intrauterine devices, contraceptives, or condoms) during the study and for 3 months after the last dose of study drug; male subjects with female partners of childbearing potential should be surgically sterile or agree to use effective contraceptive methods during the study and for 3 months after the last dose of study drug;

11) Subjects are willing to participate in this study, sign the ICF, have good compliance and cooperate with the follow-up.

4.2 Exclusion Criteria

1) Patients with metastases to the central nervous system;
2) Patients with a history of any active autoimmune disease or autoimmune disease (including, but not limited to: interstitial pneumonia, uveitis, enteritis, hepatitis, hypophysitis, vasculitis, myocarditis, nephritis, hyperthyroidism, or hypothyroidism [can be allowed after hormone replacement therapy]); patients with vitiligo or childhood asthma which has completely resolved without any intervention indicated in adulthood are allowed to be included; patients requiring medical intervention with bronchodilators are not allowed to be included;
3) Patients with congenital or acquired immune deficiencies, such as human immunodeficiency virus (HIV) infection, active hepatitis B (HBV DNA ≥ 500 IU/ml), hepatitis C (hepatitis C antibody positive and HCV-RNA above the lower limit of detection), or co-infection with hepatitis B and hepatitis C;
4) Patients who have been treated with immunosuppressive drugs, excluding nasal spray and inhaled corticosteroids or physiologic doses of systemic steroids (i.e., no more than 10 mg/day prednisone or its equivalent), within 14 days prior to the first dose of study drug;
5) Patients who have received live attenuated vaccine within 4 weeks prior to the first dose or plan to receive such vaccine during the study;
6) Patients with other malignancies in the past 3 years;

7) Evidence of previous or current pulmonary fibrosis, interstitial pneumonia, pneumoconiosis, radiological pneumonia, drug-induced pneumonia, severe impairment of lung function, etc.;
8) Patients with uncontrolled hypertension (systolic blood pressure ≥ 140 mmHg or diastolic blood pressure ≥ 90 mmHg despite optimal drug therapy);
9) Patients with grade II or higher myocardial ischemia or myocardial infarction, or poorly controlled arrhythmia (including QTc interval ≥ 450 ms in males and ≥ 470 ms in females). Patients with class III-IV cardiac insufficiency by NYHA criteria, or echocardiography suggesting of left ventricular ejection fraction (LVEF) < 50%, or patients with myocardial infarction, New York Heart Association class II or higher cardiac failure, uncontrolled angina pectoris, uncontrolled severe ventricular arrhythmia, clinically significant pericardial disease, or ECG suggesting of acute ischemia or active conduction system abnormalities within 6 months before enrollment;
10) Patients with concurrent severe infection (e.g., requiring IV antibiotics, antifungal or antiviral drugs) within 4 weeks prior to the first dose, or fever of unknown origin > 38.5 °C at screening/prior to the first dose;
11) Patients with a known history of allogeneic organ transplantation or allogeneic hematopoietic stem cell transplantation;
12) Pregnant or lactating women; or women of childbearing potential who are unwilling or unable to take effective contraceptive measures;
13) Patients who are known to be allergic, hypersensitive to or intolerant of camrelizumab, nab-paclitaxel, or their excipients;
14) Patients who are participating in other clinical studies or whose first dose day is less than 4 weeks (or 5 half-lives of the study drug) from the end of the previous clinical study (last dose);
15) Patients with a known history of psychotropic drug abuse, alcoholism, or drug addiction;
16) Patients with any condition that, in the opinion of the investigator, may jeopardize the patient or render the patient unable to meet or perform the requirements of the study.

4.3 Withdrawal Criteria

1) The subject may withdraw consent and request withdrawal;
2) Radiographic evidence of disease progression;

3) After study drug is down-titrated to the lowest dose, the subject still can not tolerate it and needs to reduce the dose;
4) Other conditions in which withdrawal from the study is deemed necessary by the investigator.
5) Lost to follow-up;
6) Death of subject.

4.4 Removal Criteria

1) Subjects who take prohibited drugs during the study;
2) Subjects who are found to have violated the inclusion and exclusion criteria after enrollment;
3) Subjects with other serious protocol violations that, in the opinion of the investigator, affect the evaluation of efficacy and safety.

4.5 Discontinuation Criteria

Discontinuation criteria for this study include, but are not limited to, the following:

1) Subjects withdraw ICF and request withdrawal (unwilling to undergo subsequent follow-up);
2) Unexpected, significant, or unacceptable risks to subjects are found;
3) The sponsor decides to discontinue the study due to reasons such as severe delays in subject enrollment or frequent protocol deviations.
4) Subject dies;
5) Subject is lost to follow-up.

5 Study Drug

5.1 Name: Camrelizumab

Manufacturer: Suzhou Suncadia Biopharmaceuticals Co., Ltd.

Dosage form: Lyophilized powder

Strength: The product is tentatively formulated as 200 mg and packaged in a 20 mL vial

Batch No.: See certificate of analysis for details

Route of administration: Intravenous drip

Shelf life: 24 months

Storage conditions: Sealed, protected from light and stored in a refrigerator at 2 to 8°C. The product should not be frozen

5.2 Name: Nab-paclitaxel

Manufacturer: Jiangsu Hengrui Pharmaceuticals Co., Ltd.

Dosage form: Lyophilized powder

Strength: 100 mg

Batch No.: See certificate of analysis for details

Route of administration: Intravenous drip

Shelf life: 12 months

Storage conditions: Protected from light and stored at 20°C to 30°C

5.3 Name: Cisplatin/carboplatin/nedaplatin for injection

For cisplatin for injection, the investigator shall select the manufacturer and strength of the drug according to the routine practice in the hospital. The manufacturer of cisplatin for injection is not specifically limited in this study; however, the lyophilized powder of cisplatin of Qilu Pharmaceutical (Hainan) Co., Ltd. (strength: 10 mg) is preferred for use in this study.

Carboplatin or nedaplatin for injection will be determined by the investigator according to routine practice of the hospital, and no special restrictions are made in this study.

5.4 Preservation and Management of Drugs

The investigator or his/her authorized personnel (e.g., pharmacist) is responsible for ensuring that all study drugs are stored under required storage conditions in an access-controlled safe area in accordance with applicable regulatory requirements.

The study drug should be stored in accordance with the storage conditions and should be stored in its original container, which should be consistent with that on the drug label. If the storage conditions on the label are inconsistent with those in other materials of the study drug (e.g., the Investigator's Brochure), the storage conditions on the label should prevail.

The study site needs to record daily maximum and minimum temperatures for all storage locations (eg, frozen, refrigerated or room temperature). The recording period should begin with receipt of study drug until the last subject completes the last visit. Even if there is a continuous monitoring system, the study site should also have a record log to ensure the correct storage temperature. Temperature monitoring devices and storage devices (e.g., refrigerators) should be checked regularly to ensure proper operation.

Once any deviation from the conditions on drug label is found, it should be promptly

reported. The study site should take active measures to transfer the study drug to a place under specified storage conditions on the label as soon as possible, and report the temperature deviation and the measures taken to the sponsor.

The study drug affected by temperature deviations should be temporarily separated and should not be used until sponsor's permission. Use of affected study drug without the sponsor's permission is a protocol deviation. The sponsor will provide the study site with the specific steps for reporting of temperature deviations.

During dosing at home, study site personnel will instruct subjects to properly store the study drug in accordance with the storage conditions of the drug.

The study drug is not allowed for treatments other than those in this study.

5.5 Drug Preparation

The study drug should be prepared by qualified or experienced study personnel (e.g., study nurse) according to the drug manual and the marketed package insert.

5.6 Management, Dispensing and Recovery of Drugs

The management, dispensing and recovery of the study drug is the responsibility of a person designated by the investigator. The investigator must ensure that all study drugs are used only for subjects participating in the clinical study, and that the dose and administration of the study drug comply with the protocol. The remaining drugs or expired drugs shall be returned to Jiangsu Hengrui Pharmaceuticals Co., Ltd. for destruction, and the clinical drugs shall not be transferred to any non-clinical study participants.

The drug receipt form must be signed by two persons at the time of receipt and kept by the study site. When the remaining drug is recovered, both parties will sign the drug recovery form. The dispensing and recovery of each study drug should be recorded in a timely manner on a special record sheet.

6 Dose Regimen

6.1 Randomization and Dose

Ninety-four patients will be enrolled, including 47 patients in the group with camrelizumab and 47 patients in the group without camrelizumab

- Camrelizumab: A fixed dose of 200 mg will be administered by intravenous drip over 30 minutes (the overall infusion time, including flush time, is not less than 20 minutes and not more than 60 minutes) on Day 1 of each cycle, every 3 weeks as a

cycle.

- Nab-paclitaxel: 130 mg/m^2 by intravenous drip over 30 minutes on Day 1 and 8 of each cycle, every 3 weeks as a cycle.
- Cisplatin/carboplatin/nedaplatin:

  Cisplatin: 75 mg/m^2 by intravenous drip on Day 1 of each cycle, every 3 weeks as a cycle.

  Or carboplatin: AUC5 by intravenous drip on Day 1 of each cycle, every 3 weeks as a cycle.

  Or nedaplatin: 100 mg/m^2 by intravenous drip on Day 1 of each cycle, every 3 weeks as a cycle.

Camrelizumab in combination with nab-paclitaxel and cisplatin/carboplatin/nedaplatin or nab-paclitaxel in combination with cisplatin/carboplatin/nedaplatin as neoadjuvant therapy for 3 cycles, off for 4 to 6 weeks, and then followed by surgery for the treatment of resectable NSCLC.

6.2 Dose Interruption and Modification

6.2.1 General Principles for Dose Modification

Checkpoint inhibitors and chemotherapy have different mechanisms of action and differ greatly in toxicity profiles, and there is currently no evidence to suggest a significant interaction. There are also some differences in the toxicity profiles of paclitaxel and platinum. Therefore, if the investigator judges that the toxicity is attributed to some of the administered drugs and not attributed to other drugs, it is possible only to adjust the dose of those drugs that are associated with the toxicity, or interrupt/delay/terminate the treatment with those drugs related to the toxicity. If it is not possible to clearly attribute the toxicity to any drugs, it is necessary to adjust all the administered drugs. The efficacy of anti-tumor drugs is continuous, and there is no evidence of significant reduction in efficacy for the treatment interruption/delay within a specified time period. Therefore, if only the treatment of drugs related to toxicity is interrupted/delayed for a subject, all drugs will be given as initially planned when the toxicity is substantially resolved, thus avoiding disrupting the combination treatment. Efforts should be made to ensure all drugs are given within the prescribed time window of each cycle.

For example, subjects in the treatment group are found to have serious platelet count decreased prior to dosing on C2D1, it is judged to be related to chemotherapy and unrelated to camrelizumab, the treatment is interrupted and symptomatic treatment is given as specified in the protocol, and close reexamination is performed. If the platelet count on C2D2 to D3 has returned to the protocol-required level, the three drug therapies may be continued sequentially at the time of recovery, with the subsequent time window unchanged; if the platelet count on C2D4 to D7 has returned to the protocol-required level, the three drug therapies may be continued sequentially at the time of recovery, and the subsequent time window needs to be recalculated; if the platelet count has not recovered on C2D7, the treatment with camrelizumab can be continued, and the subsequent time window needs to be recalculated; during which if it is found that the platelet count has recovered on C2D15, the chemotherapy should also not be administered, and the three drug therapies should be continued sequentially until the scheduled time of C3.

6.2.2 Dose Interruption and Modification of Camrelizumab

The dose of camrelizumab is not allowed to be increased or reduced, and if a subject delays or interrupts treatment with camrelizumab due to toxicity, the treatment may continue at the original dose when the toxicity improves within the specified time frame. Dose delay/interruption is allowed up to 12 weeks calculated from the date when the last dose is administered before the treatment is discontinued. The time window for each dose is calculated starting from the date of the first dose. Dose delay is generally no more than 3 days, unless appropriate reasons are specified. If a dose is delayed for 3 days but less than 7 days, the actual dosing time is recommended to be used for the calculation of the time window of next dose. Subsequent dosing time window thereafter is still within 3 days. If a dose is delayed for more than 7 days, the dose should not be made up for the cycle. And the dosing of next cycle should be continued as scheduled, with the dosing time window calculated based on the first dose.

6.2.3 Dose Interruption and Modification of Nab-Paclitaxel

Dose modification of nab-paclitaxel is permitted. The dose of nab-paclitaxel can be sequentially down-titrated to 75% and 50% of the original dose, i.e. 100 mg/m^2 and 65

mg/m^2, respectively, depending on tolerability. For dose delays due to continued Grade 3 drug-related thrombocytopenia, neutropenia > 7 days, or any other reasons, the scheduled dose should be skipped. Doses skipped or missed will not be replaced. If the chemotherapy regimen (paclitaxel + cisplatin/carboplatin/nedaplatin) is discontinued for more than 6 weeks and the requirements for chemotherapy still cannot be met, the chemotherapy should be permanently discontinued. Patients should be withdrawn from the study.

The time window for each dose is calculated starting from the date of the first dose. Dose delay is generally no more than 3 days, unless appropriate reasons are specified. If a dose is delayed for 3 days but less than 7 days, the actual dosing time is recommended to be used for the calculation of the time window of next dose. Subsequent dosing time window thereafter is still within 3 days. If a dose is delayed for more than 7 days, the dose should not be made up for the cycle. And the dosing of next cycle should be continued as scheduled, with the dosing time window calculated based on the first dose.

6.2.4 Dose Interruption and Modification of Cisplatin/Carboplatin/Nedaplatin for Injection

There is no requirement for dose interruption and dose modification of cisplatin/carboplatin/nedaplatin for injection in this study. The investigator shall make appropriate modifications according to the package insert, clinical practice and the patient's personal condition.

6.3 Concomitant Medications and Treatments

Concomitant medications are defined as the drug/treatment other than the study treatment, given at the discretion of the investigator and in the interest of the subjects. Concomitant medications, blood products and non-drug interventions (e.g., aspiration) received by subjects from 30 days prior to the first dose of study drug to the end of the study safety visit should be recorded in the case report form in strict accordance with GCP requirements.

Drugs or vaccines explicitly prohibited in the protocol are prohibited throughout the study. If a subject develops a concomitant disease that must use the prohibited drug, it may be necessary to discontinue the study drug or receive a prohibited drug. The

investigator should discuss with the sponsor whether the subject should continue the treatment with study drug or receive the prohibited drug, and the final decision should be made jointly by the investigator and the subject.

All patients are given the planned therapeutic dose of the drug, and dose interruption may be considered based on the worst hematological or other toxicities, if necessary.

6.3.1 Other Anti-tumor Therapies or Investigational Drugs

When a subject is on study treatment, other anti-tumor therapies not specified in this protocol will not be allowed, including modern herbal products preparations approved for marketing by the CFDA indicated for anti-tumor therapy, and immunomodulatory agents (including but not limited to: interferon, interleukin-2, thymosin, etc.).

Participation in clinical studies of other drugs/devices is not allowed.

Other systemic anti-tumor therapies, such as chemotherapy, molecular targeted therapy, hormonal therapy, immunotherapy, biotherapy and radiation therapy, are not allowed.

6.3.2 Vaccines

Activated attenuated vaccines should not be used within 4 weeks prior to the first dose and during the study. Subjects who are expected to require the use of an activated attenuated vaccine during the study are not recommended for enrollment.

Vaccines used to prevent infectious diseases, such as pneumonitis, influenza vaccines, are allowed, but must be inactivated vaccines. Other vaccines must be discussed with the sponsor before use.

6.3.3 Immunomodulatory Agents and Corticosteroids

Concurrent immunosuppressive therapy (except for the management of drug-related AEs) is not allowed.

Concurrent treatment with immunostimulants (except for the management of drug-related AEs) is not allowed.

Long-term, systemic use of corticosteroids is not allowed. Cumulative use of corticosteroids as specified in the package insert for ≤ 1 week as premedication for chemotherapy or contrast agent allergy is allowed. Systemic corticosteroid therapy is allowed for individual subjects after discussion. Short-term (no more than 3 weeks) use of corticosteroids for non-autoimmune diseases (e.g., delayed allergic reactions caused

by contact allergens) is allowed.

However, emergency use, topical application, inhalation by spray, eye drops, or local injection of corticosteroids is allowed. Systemic corticosteroids (≤ 10 mg/day Prednisone or equivalent) at physiologic replacement doses (e.g., adrenal replacement steroid doses) are allowed.

6.3.4 Hematopoietic Growth Factors and Blood Transfusion

The use of hematopoietic stimulating factors such as granulocyte colony-stimulating factor, erythropoietin, and thrombopoietin, as well as blood transfusion and blood products (such as albumin, etc.) as primary prevention before treatment is not allowed, but is allowed for the treatment of adverse events (AEs).

6.3.5 Anti-inflammatory Therapy

Anti-inflammatory or narcotic analgesics may be administered if there are no known or predictable drug interactions and they are not prohibited medications as specified in the protocol.

6.3.6 Medications to be Used with Caution during the Study

- Coumarins (high plasma protein binding):

Drugs (such as warfarin) have high plasma protein binding, which may affect the plasma concentration of study drug.

Paclitaxel is metabolized by CYP3A4 and CYP2C8, and the following drugs may interact with paclitaxel and affect the metabolism of paclitaxel.

- CYP3A4 and CYP2C8 inhibitors:

Imidazole antifungals (such as ketoconazole and fluconazole), erythromycin, fluoxetine, nefazodone, quercetin, gemfibrozil, quinidine, testosterone, 17α-diethylstilbestrol, retinoic acid, etc.;

- CYP3A4 and CYP2C8 inducers:

Rifampicin, carbamazepine, phenytoin, fructus forsythiae, etc.

7 Follow-up

After the end of treatment (i.e., disease progression, or intolerable adverse reactions), or after withdrawal/discontinuation of treatment, all subjects will be followed until the subject dies or is lost to follow-up.

8 Clinical Data Collection

8.1 Baseline Examinations

Informed consent will be signed and medical history, infectious disease screening (hepatitis B, hepatitis C, syphilis, AIDS), tumor lesion imaging (CT or MRI) data, pathological assessment, pulmonary function assessment, AEs and concomitant medication records will be collected within 14 days prior to treatment. Eligibility criteria and hematology, blood chemistry, ECOG score, vital signs, physical examination, urinalysis, stool routine, coagulation, thyroid function test, 12-lead ECG, echocardiography, pregnancy test, AEs and concomitant medication record will be checked within 7 days prior to treatment.

8.2 On-treatment Examinations

Vital signs, physical examination, PS score will be performed before treatment on Day 1 of Cycle 1; vital signs, physical examination, PS score, hematology, urinalysis, stool routine, blood chemistry, coagulation function test, thyroid function test, 12-lead ECG, immune marker test will be performed and AEs and concomitant medications will be recorded in Cycle 2 and Cycle 3.

8.3 Preoperative Examinations

After the end of the treatment cycle, vital signs, physical examination, PS score, hematology, urinalysis, stool routine, blood chemistry, coagulation, thyroid function test, 12-lead ECG, pregnancy test, tumor lesion imaging (CT or MRI) data, pulmonary function assessment, AEs and concomitant medications will be recorded 7 days before operation.

8.4 End of Study Treatment/Withdrawal from the Study

At the end of study treatment or withdrawal from the study, if the patient does not have an examination within 7 days prior to the end of study, the following tests should be performed: vital signs, physical examination, PS score, urinalysis, hematology, blood chemistry, stool routine, thyroid function test, 12-lead ECG, pregnancy test, pathological assessment, AEs and concomitant medication recording.

8.5 Follow-up

Safety follow-up: it will be performed every 30 days (± 3 days) from the last study treatment until 90 (± 7 days) after the last study treatment. The first safety visit should

be conducted at the study site, and safety assessments and follow-up of AEs should be performed, and concomitant treatment should be recorded. This follow-up should include the following items: vital signs, physical examination, PS score, urinalysis, hematology, blood chemistry, stool routine, thyroid function test, 12-lead ECG, pregnancy test, tumor lesion imaging (CT or MRI) data, AEs and concomitant medication records. Subsequent safety follow-up will be conducted by telephone, and only survival information, concomitant medications/therapies and AEs need to be collected.

Survival follow-up: after the end of the safety follow-up period, the subject will enter the survival follow-up visit to collect disease progression (date of progression) and subsequent anti-tumor therapy until the subject's death, lost to follow-up, withdrawal of informed consent or study termination by the sponsor. During this period, the subjects will be followed every 3 months (± 7 days) in the first and second years, every 6 months (± 7 days) from the third year to the fifth year, and annually thereafter to collect survival and subsequent treatment information.

8.6 Unscheduled Visits

Subjects may need to undergo unscheduled visits in the course of the study, and the follow-up should include the following items:

- Adverse event;
- Concomitant medication/treatment;
- Relevant examinations (including imaging, if any).

9 Efficacy Evaluation Variables

9.1 Primary Efficacy Variable

Pathological Complete Response (pCR) rate:

defined as the proportion of patients with no residual tumor cells in the surgically resected tumor sample and all sampled regional lymph nodes after neoadjuvant treatment.

9.2 Secondary Efficacy Variables

Major pathological response (MPR) rate:

defined as the proportion of patients with ≤ 10% of residual tumor cells in the surgically resected tumor sample and sampled regional lymph nodes after neoadjuvant treatment.

Objective response rate:

defined as the proportion of patients (including cases of radiographic complete response [CR] and partial response [PR]) whose tumor size shrinks to predefined values prior to definitive surgery. Objective tumor response will be assessed using RECIST 1.1. Subjects must have measurable tumor lesions at baseline, and the response evaluation criteria are classified as CR, PR, stable disease (SD), and progressive disease (PD) according to RECIST 1.1.

Event-free survival (EFS):

defined as the time from the randomization to the first occurrence of disease progression or recurrence, or death (due to any cause).

9.3 Exploratory Efficacy Variable

The feasibility of surgery:

included type of surgery, R0 Resection, surgical approach, duration from final treatment to surgery, duration of surgery, length of hospital stay.

10 Safety Parameters

10.1 Adverse Event Observation

10.1.1 Definition of Adverse Events

An AE is defined as any untoward medical occurrence in a patient or clinical study subject administered a drug or received a treatment regimen, which does not necessarily have a causal relationship with this treatment.

An AE can therefore be any unfavorable and unintended sign (including an abnormal laboratory finding), symptom, or disease temporally associated with the use of a medicinal product, whether or not considered related to the medicinal product.

Events that occur during the pre- and post-treatment phase are also considered AEs according to applicable regulations. Therefore, safety monitoring of AEs or serious adverse events (SAEs) should be reported from the time the subject enters the study (signing the informed consent form and eligible for screening) to 90 days after the last treatment.

10.1.2 Adverse Event Grading

AEs will be graded into 1-5 grades according to NCI Common Acute and Subacute Toxicity Grading Criteria (NCI-CTCAE 5.0). AEs not listed in the NCI toxicity grading criteria can be judged according to the following criteria:

Grade 1 (mild): feeling uncomfortable but not affecting normal daily activities;

Grade 2 (moderate): marked discomfort, but the extent of discomfort is not sufficient to reduce or interfere with normal daily activities;

Grade 3 (severe): inability to work or carry out normal daily activities;

Grade 4 (fatal): disabling or fatal.

10.1.3 Determination of Relationship between Adverse Events and Study Drug

1) Related: consistent with the known type of reactions of the drug used; consistent with a reasonable chronological order after administration; alleviation or disappearance of the AE after dose reduction or drug withdrawal, and re-occurrence after re-administration.
2) Probably related: consistent with the known type of reactions of the drug used; consistent with a reasonable chronological order after administration; alleviation or disappearance of the AE after dose reduction or drug withdrawal, but the event can also result from patient's clinical status or other reasons.
3) Unlikely related: not so consistent with the known type of reactions of the drug used; not so consistent with a reasonable chronological order after administration; the event can also result from patient's clinical status or other reasons.
4) Not related: not consistent with the known type of reactions of the drug used; not consistent with a reasonable chronological order after administration; the event can also be explained by patient's clinical status or other reasons; the event is alleviated or disappears after the clinical symptoms or other reasons are excluded.
5) Not evaluable: the relationship between the drug used and the adverse reaction cannot be explained.

Table 9 Determination of Relationship between Adverse Events and Study Drug:

Grade	Clinical Description of Severity
1	Mild; asymptomatic or mild symptoms; only clinical or laboratory abnormalities; intervention not indicated
2	Moderate; requiring minimal, local or noninvasive treatment; limited age-appropriate instrumental activities of daily living (ADL) (instrumental ADL refer to cooking, shopping, making telephone calls, managing money, etc.)
3	Severe or medically significant but not immediately life-threatening;

	hospitalization or prolongation of existing hospitalization indicated; leading to disability; limiting self-care ADL. Self-care ADL refers to bathing, dressing and undressing, feeding self, using the toilet, taking medications, and not bedridden
4	Life-threatening consequences; urgent intervention indicated
5	Leading to death

10.2 Peri- and post-operative complications

Surgery related events reported up to 30 days after surgery. The Clavien-Dindo scale classifies peri- and post-operative complications into 7 grades.

Grade I: Abnormal changes requiring antiemetics, antipyretics, painkillers, electrolytes, and physical therapy without surgery, endoscopy, or radiation, but including wound infections requiring open drainage;

GradeII: Blood transfusion, total parenteral nutrition, and drug therapy in addition to Grade I drugs are required;

Grade IIIa: Surgery, endoscopy, radiotherapy and other interventions are not required under general anesthesia;

Grade IIIb: Surgery, endoscopy, radiotherapy and other interventions are required under general anesthesia;

Grade IVa: life-threatening complications, single organ dysfunction (including dialysis);

Grade IVb: life-threatening complications, multiple organ dysfunction;

Grade V: Death.

10.3 Serious Adverse Event

If SAEs occur during the course of the study, the investigator should immediately take appropriate protective measures to the subject and report to the principal investigator within 24 hours. The investigator should complete the SAE Report Form, sign and date the report. SAEs include:

1) Death or life threatening;
2) Hospitalization or prolongation of hospitalization
3) Permanent disability

4) Carcinogenesis
5) Teratogenicity;
6) Overdose;

10.4 Immune-mediated Adverse Events

Immune-mediated adverse events (IMAEs) are defined as specific events (including: pneumonia, diarrhea/colitis, hepatitis, nephritis/kidney dysfunction, rash, and endocrine lesions) for which the subject receives immunosuppressive drugs. Endocrine events (thyroid function decreased/thyroiditis, hyperthyroidism, hypophysitis, diabetes mellitus, adrenal insufficiency) are generally exceptional, because they are generally unrelated to the treatment and can usually be managed without immunosuppressive intervention. PT included in the IMAE analysis to support warnings and precautions are shown in the table below.

Table 10 Preferred Terms Included in IMAE Analysis to Support Warnings and Precautions

IMAE Category	PT Included under IMAE Category (MedDRA Code)
Pneumonia	Pneumonia, interstitial pneumonia
Diarrhea/Colitis	Diarrhea, colitis, enterocolitis
Hepatitis	Hepatotoxicity, hepatitis, acute hepatitis, autoimmune hepatitis, AST increased, ALT increased, bilirubin increased, ALP increased
Adrenal insufficiency	Adrenal insufficiency
Thyroid function decreased/Thyroiditis	Acute thyroiditis (prostration with frequent thyroiditis), autoimmune thyroiditis (prostration with frequent thyroiditis)
Hyperthyroidism	Hyperthyroidism
Hypophysitis	Hypophysitis
Diabetes mellitus	Diabetes mellitus, diabetic ketoacidosis
Nephritis and renal dysfunction	Nephritis, acute nephritis, tubulointerstitial nephritis, acute renal failure, renal failure, creatinine increased
Rash	Rash, rash maculo-papular

10.5 Potential Drug-induced Liver Injury

1) Drug-induced liver injury will be considered if AST and/or ALT levels are abnormal and total bilirubin levels are abnormally increased, the following conditions are met and there are no other causes of liver injury. Such situations should always be considered medically significant events and should be reported as SAEs.
2) Subjects should return to the study site for assessment as soon as possible (preferably within 48 hours) after awareness of the abnormal results. Assessments should include laboratory tests, detailed medical history and physical assessments, and the possibility of liver tumor (primary or secondary) should be considered.
3) In addition to repeated testing of AST and ALT, laboratory test items should also include albumin, creatine kinase, total bilirubin, direct bilirubin and indirect bilirubin, gamma glutamyltransferase, prothrombin time (PT)/international normalized ratio (INR), and alkaline phosphatase. Detailed medical history should include alcohol use, acetaminophens, soft drugs, supplements, family's medical history, occupational exposure, sexual activity, travel history, contact with jaundiced patients, surgery, blood transfusion, liver disease, or allergic disease history, etc. Further tests may include testing for acute hepatitis A, B, C, and E and imaging of liver (such as biliary tract). If repeated testing of the above items confirms that the definition of the laboratory criteria described above is met, the possibility of potential drug-induced liver injury should be considered in the absence of other causes of abnormal liver function, without waiting for the results of all etiological tests of liver function. Such cases of potential drug-induced liver injury should be reported as SAEs.

10.6 Observation and Management of Adverse Events

10.6.1 Management of Adverse Reactions and Adverse Events

The study ward should be equipped with emergency drugs, oxygen, sphygmomanometers, electrocardiographs, defibrillators, ventilators, and drugs and equipment required for routine infusion. The subject should receive drugs under the supervision of a clinical physician, and the subject's vital signs and other adverse

reactions should be observed until the end of the study. Symptomatic treatment should be given to subjects with different situations of AEs and SAEs, and necessary rescue measures may be taken in emergency situations.

10.6.2 Management Principles of Adverse Reactions

In general, after minor adverse reactions occur, condition observation shall be performed firstly to make a definite diagnosis and judge the prognosis. If it does not affect the study, measures such as observation and symptomatic treatment may be taken to control the adverse reactions of the subject as much as possible, so as to make the adverse reactions improve, disappear or stabilize, and do not continue to worsen.

10.6.3 Management of Adverse Events (including but not limited to the following possible therapeutic measures)

1) Special management
    a) Dose interruption.
    b) Monitoring vital signs: electrocardiogram, blood pressure, respiration, body temperature.
    c) Gastric lavage: 1%-2% sodium chloride solution or 1:5000 potassium permanganate solution.
    d) Catharsis: sodium sulfate 15-30 g in 200 mL of water.
    e) Enema: continuous washing with 1% mildly warm soapy water (about 5000 mL) in a high position.
2) Supportive care:
    a) sedation, oxygenation (as appropriate).
    b) Establishing an intravenous infusion channel, open the respiratory channel, and, if necessary, providing endotracheal intubation, extracardiac massage, and ventilator-assisted respiratory support.
    c) Adequate hydration to maintain circulating blood volume: intravenous injection of normal saline or glucose and sodium chloride injection, supplementing colloid according to the condition to ensure the circulating osmotic pressure.
    d) Cardiotonic and pressor measures may be taken if necessary to maintain blood pressure stabilization and ensure the blood supply of important organs: adrenocorticoid 10-60 mg plus 50-250 mL of 5% glucose injection can be given intravenously at first, and dopamine can be

pumped for maintenance after blood pressure is stable.

e) Diuretics: diuretics such as furosemide may be given according to urine volume, and sodium bicarbonate may be given in an appropriate amount to alkalize urine.

f) Maintaining water-electrolyte, acid-base balance.

g) If arrhythmia occurs, symptomatic antiarrhythmic therapy should be given.

h) Symptomatic treatment, nutritional support.

3) Anti-allergy (if necessary):

   a) Promethazine 25-50 mg intramuscularly, keep supine with oxygen inhalation to ensure airway patency.

   b) 0.1-0.2 mL of 0.1% epinephrine may be given subcutaneously or with 5% glucose intravenously.

   c) Glucocorticoids, such as dexamethasone 5-10 mg IV, may be used.

4) Prophylactic gastric acid inhibitors, omeprazole 40 mg PO or IV.

5) Correcting respiratory failure circulatory: oxygenation or artificial respiration with alternative injection of coramine 0.375 g and lobeline 3-6 mg every 15-30 minutes, and intravenous injection 1-2 times, and using cardiac glycoside digitalis preparations if necessary.

10.7 Follow-up and Reporting of Adverse Events

10.7.1 Follow-up of Adverse Events/Serious Adverse Events

All AEs/SAEs, whether related or not related to the study drug (from signing the ICF until 4 weeks after the last dose of the study drug), should be reported. Only SAEs considered related to the study drug will be reported 4 weeks after the last dose. All AEs/SAEs should be followed until the event disappears, resolves to baseline or ≤ Grade 1, reaches a stable state, or can be reasonably explained (e.g., lost to follow-up, death), and related information should be recorded timely and accurately in the CRF.

10.7.2 Reporting of Adverse Events

AE information should be collected from the time the subject signs the ICF until the first visit (within 4 weeks) after the last dose. At each visit, the investigator should inquire about AEs that occurred after the last visit. The investigator should follow up until the AE disappears, resolves to baseline or ≤ Grade 1, reaches a stable state, or can

be reasonably explained (lost to follow-up, death); and the information should be recorded timely and accurately in the CRF.

10.7.3 Reporting of Serious Adverse Events

SAEs should be collected from the time the subject signs the ICF until 4 weeks after the last dose of the study drug (including the last day of 4 weeks). In the event of an SAE, the investigator must immediately complete the Serious Adverse Event Report Form, sign and date the report within 24 h of awareness, and immediately submit it to the Ethics Committee office and the project leader of Jiangsu Hengrui Pharmaceuticals Co., Ltd.

SAEs occurring after 90 days after the last dose of the study drug will generally not be reported unless they are suspected to be related to the study drug. For SAEs, the symptoms, severity, relationship to study drug, time of occurrence, time of management, measures taken, follow-up time and manner, and outcome should be recorded in detail. If the investigator considers that an SAE is unrelated to study drug but potentially related to the study conditions (e.g., termination of the pre-existing treatment, or comorbidities during the trial), details should be described in the narrative section of the SAE report. If the severity of an ongoing SAE or its relationship to study drug is changed, a follow-up report should be submitted immediately. False information included in the previously SAE report per the investigator may be corrected, revoked or downgraded in the follow-up report, and reported in accordance with the SAE reporting procedures.

10.7.4 Reporting of Adverse Events of Special Interest

If it is also an SAE, please complete the CFDA Serious Adverse Event Report Form at the same time and report it to the relevant unit according to the SAE reporting procedure.

10.7.5 Reporting of Pregnancy

If a female subject becomes pregnant during the clinical study, the subject should immediately discontinue study drug and complete the Pregnancy Report/Follow-up Form within 24 h of the investigator becoming aware of the pregnancy.

If the partner of a male subject becomes pregnant during the clinical study, the subject can continue the clinical study and should complete the Pregnancy Report/Follow-up

Form within 24 h of the investigator becoming aware of the pregnancy.

The investigator will follow up the pregnancy outcome until 1 month after delivery.

Pregnancy outcomes of stillbirth, spontaneous abortion or fetal malformation will be considered as SAEs, and should be reported within specified time limit for SAE reporting.

11 Data Management

In this study, CRFs will be used for the collection and management of study data. At the time of data completion, the data in the CRF should be derived from original documents such as the original medical records and laboratory test reports, and all data should be consistent with the original documents. Any observations and examination results in the study should be timely, correct, complete, clear, standardized and authentic. If data correction is required for the CRF, fill in the reason for the data modification. In addition, the investigator should complete, review and submit the CRF within 5 working days after the end of each subject's treatment course. The investigator or data entry personnel (CRC) should promptly respond to queries from monitors, data managers, and medical reviewers. Upon completion of data cleaning, the investigator should sign and confirm the completed CRF.

After the completion of the clinical study, the study director, statistical experts and data manager will jointly review the data before statistical analysis.

12 Statistical Analysis

12.1 Statistical Hypothesis

The null hypothesis H_0: $pCR_{treatment} = pCR_{control}$ will be tested using a 2-sided, 5% alpha level, against the alternative hypothesis H_a: $pCR_{treatment} \neq pCR_{control}$.

The null hypothesis is to be rejected at a two-sided significance level of 0.05.

12.2 Analysis Sets

- Full Analysis Set (FAS) is a modified ITT population to include all randomized subjects who received at least 1 dose of study treatment. FAS will serve as the main analysis set of efficacy analysis.

- Surgical Evaluable Set (SES) will include all randomized subjects who received at least 1 dose of study treatment and received surgery.
- Safety Analysis Set (SAS): including all enrolled subjects who have taken at least one dose of the study drug.

12.3 General Statistical Considerations

All statistical analyses will be performed using statistical analysis software SAS® Version 9.4. Descriptive statistics including number of subjects with non-missing values, mean, standard deviation, median, minimum, and maximum, will be presented for continuous variables. For categorical variables, frequencies with percentages will be presented. Time-to-events variables will be analyzed by using Kaplan-Meier method and Kaplan-Meier plots will be presented.

12.4 Analysis of Primary Efficacy Endpoints

FAS will be used as the primary analysis set for the primary efficacy analysis. pCR is defined as number of subjects with an absence of viable tumor cells in the surgical specimens from the primary tumor and all sampled regional lymph nodes divided by number of subjects who received at least one dose of study treatment.The number and proportion of subjects with pCR in each treatment group will be presented. The 95% confidence interval of pCR will be estimated by using Clopper-Pearson method. A two-sided 95% confidence interval for odds ratio of pCR between the treatment groups will also be computed. Fisher's exact test will be used to test the difference of pCR between treatment groups.

12.5 Analysis of Secondary Efficacy Endpoints

For binary secondary efficacy endpoints, including MPR rate and ORR, the number and proportion of subjects in each treatment group will be presented. The 95% confidence interval will be estimated by using Clopper-Pearson method. A two-sided 95% confidence interval for odds ratio between the treatment groups will also be computed. For time-to-event secondary efficacy endpoints, the Kaplan-Meier method will be used to estimate the distribution of EFS in each treatment group, the 95% confidence intervals of median EFS will be calculated by using Brookmeyer and

Crowley method, and the 95% confidence intervals of EFS rates by complementary log-log method. The corresponding Kaplan-Meier plots will also be presented. In addition, the unstratified Cox proportional hazards regression model will be used to estimate the hazard ratio and the associated 95% confidence interval.

12.6 Safety Analyses

All the safety analysis will be performed for SS except for surgery-related adverse events will be analyzed in SES.

Safety endpoints include treatment emergent adverse events (TEAE), treatment related adverse events (TRAE), serious adverse events (SAE), adverse events leading to treatment discontinuation, interruption or dose reduction, immune-mediated adverse events, and death.

AE will be summarized by treatment groups. An AE will be considered as a TEAE occurred or becomes worse in severity after the initiation of study treatment and within 90 days after last study treatment administration. If the CTCAE grade of an AE is missing, it will be considered as grade 3 AE. The TEAEs which being recorded as being “related with”, “highly-possibly related with” and “possibly related with” study treatment will be considered as treatment related TEAE (TRAE). If the relationship to the study treatment is missing, it will be considered to be related to study treatment. A high-level summary of the number of subjects with TEAEs will be presented by the treatment groups, including the number and percentage of subjects with any TEAEs, TEAEs with CTCAE grade ≥ 3, TEAE leading to dose reduction, TEAE leading to treatment interruption, TEAE leading to treatment discontinuation, TEAE leading to death, Serious TEAE (TESAE), immune-mediated TEAE, Treatment related TEAEs (TRAEs), TRAEs with CTCAE grade ≥ 3, TRAEs leading to dose reduction, TRAEs leading to treatment interruption, TRAEs leading to treatment interruption, TRAEs leading to death, serious TRAEs, or immune-mediated TRAEs. Surgery-related adverse events will be analyzed in SES. Number and percentage of subjects with

surgery related adverse events for any grade and grade$\geq$3 will be summarized by the treatment groups.

13 Protection of Subject's Rights and Interests

The ethics committee and informed consent form are the main institution and measure to protect the rights and interests of subjects. Prior to the start of the clinical study, the protocol must be reviewed, approved and signed by the Ethics Committee. During the conduct of the clinical study, any amendments to the protocol should be approved by the ethics committee before implementation.

The clinical investigator must explain to the subject that participation in the clinical study is voluntary, he/she has the right to withdraw from the study at any time at any phase of the study without being discriminated against or retaliated, and his or her medical treatment and interests will not be affected, and may continue to receive other treatments. Subjects must be made aware that their participation and personal data in the study are confidential. Subjects should also be informed of the nature of the clinical study, study objectives, expected possible benefits, possible risks and inconveniences, other treatment options available to them, and the rights and obligations of subjects in accordance with the Declaration of Helsinki, etc. so that they have sufficient time to consider whether to participate in the study and sign the informed consent form.

14 References

1. NCCN Clinical Practice Guidelines in Oncology (NCCN Guidelines®) Non-small Cell Lung Cancer Version 2.2016.
2. McElnay P, Lim E. Adjuvant or neoadjuvant chemotherapy for NSCLC. J Torac Dis, 2014, 6 Suppl 2: S224-S227.
3. Forde PM, Chaft JE, Smith KN, et al., Neoadjuvant PD-1 Blockade in Resectable Lung Cancer. N Engl J Med. 2018 May 24;378(21):1976-1986
4. Rusch V W, Chaft J E, Johnson B, et al. Neoadjuvant atezolizumab in resectable non-small cell lung cancer (NSCLC): Initial results from a multicenter study (LCMC3)[J]. Journal of Clinical Oncology, 2018, 36, 8541. DOI: 10.1200/JCO. 2018.36.15_suppl.8541.
5. Cascone T, William W N, Weissferdt A, et al. LBA49 Neoadjuvant nivolumab (N) or nivolumab plus ipilimumab (NI) for resectable non-small cell lung cancer (NSCLC)[J]. Annals of Oncology, 2018, 29(suppl_8): mdy424. 059.
6. Provencio-Pulla M, Nadal-Alforja E, Cobo M, et al. Neoadjuvant chemo/immunotherapy for the treatment of stages IIIA resectable non-small cell lung cancer (NSCLC): A phase II multicenter exploratory study—NADIM study-SLCG[J]. Journal of Clinical Oncology, 2018, 36, 8521. DOI: 10.1200/JCO. 2018.36.15_suppl.8521.
7. Zhou et. Phase Ib study of apatinib combined with SHR-1210 (PD-1) in patients with advanced NSCLC. 2018 ASCO e21017.
8. Von Hoff DD, Ervin T,Arena FP, et al.Increased Survival in Pancreatic Cancer with nab-Paclitaxel plus Gemcitabine. N Engl J Med.2013 Oct 31; 369(18):1691-703.

Amendment History

Amendment 1: January 8, 2020 (version 2.0)

- Inclusion criteria were revised from "patients with resectable stage II-IIIB (IIIB T3N2 only) NSCLC, according to the eighth edition of the clinical TNM staging of tumors" to "patients with resectable stage IIIA-IIIB (IIIB T3N2 only) NSCLC, according to the eighth edition of the clinical TNM staging of tumors".

Amendment 2: March 23, 2020 (version 3.0)

- Baseline pituitary-adrenal axis test was deleted in Study Flow Chart section.
- Myocardial zymogram examinations prior to dosing in the second treatment cycles, before and after surgery, and at the safety follow-up visit were added.
- The time window for baseline cranial MRI was revised to "within 60 days prior to the first dose of study drug".
- Baseline thyroid function test items were revised to five items.

Amendment 3: May 20, 2020 (version 4.0)

- The section of dose interruption and modification was added, that is, dose modifications of nab-paclitaxel and platinum were permitted.

Amendment 4: September 14, 2020 (version 5.0)

- Single-center was revised to multi-center.
- The restrictions on cisplatin manufacturers were deleted.

Amendment 5: October 14, 2020 (version 6.0)

- The types of platinum agents (carboplatin and nedaplatin) were added.

STATISTICAL ANALYSIS PLAN
(SAP)

NEOADJUVANT CAMRELIZUMAB PLUS NAB-PACLITAXEL AND INVESTIGATOR'S CHOICE OF PLATINUM VERSUS CHEMOTHERAPY ALONE IN PATIENTS WITH RESECTABLE STAGE IIIA/IIIB NON-SMALL-CELL LUNG CANCER: A RANDOMIZED CLINICAL TRIAL

VERSION 1.0, 15 SEPT 2022

CONFIDENTIALITY STATEMENT

The information is provided to you in confidence to enable you to perform the work. Do not give this document or any copy of it or reveal any proprietary information contained in it to any third party or person without the prior permission of an authorization.

**DOCUMENT REVISION HISTORY**		
**Document Version**	**Document Date**	**Reason for Revision**
1.0	15-Sept-2022	First version – Not applicable

TABLE OF CONTENTS

1. INTRODUCTION 5
2. STUDY OBJECTIVES 6
2.1. Primary Objectives 6
2.2. Secondary Objectives 6
2.3. Exploratory Objectives 6
3. STUDY DESIGN AND METHODS 7
3.1. General Study Design and Plan 7
3.2. Randomization 7
3.3. Blinding 7
4. STUDY ENDPOINTS 8
4.1. Efficacy Endpoint(s) 8
4.1.1. Primary Efficacy Endpoint(s) 8
4.1.2. Secondary Efficacy Endpoint(s) 8
4.1.3. Exploratory Efficacy Endpoint(s) 8
4.2. Safety Endpoint(s) 8
5. SAMPLE SIZE DETERMINATION 9
6. GENERAL AND STATISTICAL CONSIDERATIONS 10
6.1. Analysis Sets 10
6.1.1. Full Analysis Set 10
6.1.2. Surgical Evaluable Set 10
6.1.3. Safety Analysis Set 10
6.2. Statistical Considerations and Reporting Conventions 10
6.2.1. Statistical Software 10
6.2.2. Statistical Summary Conventions 10
6.2.3. General Reporting Conventions 10
6.2.4. Subgroups 10
7. STATISTICAL ANALYSIS 12
7.1. Demographic and Baseline Characteristics 12
7.2. Efficacy Analyses 12
7.2.1. Analysis of Primary Efficacy Endpoints 12
7.2.1.1. Main Analysis of the Primary Efficacy Endpoint 12

7.2.1.2. Subgroup Analysis 12
7.2.2. Analysis of Secondary Efficacy Endpoints 13
7.2.2.1. Major Pathological Response 13
7.2.2.2. Event-free Survival 13
7.2.2.3. Objective Response Rate (ORR) 14
7.2.3. Analysis of Exploratory Efficacy Endpoints 14
7.2.3.1. Surgical Related Endpoints 14
7.3. Safety Analyses 14
7.3.1. Adverse Events 14
7.3.2. Surgery-related Adverse Events 15
8. SUMMARY OF CHANGES TO THE STATISTICAL ANALYSES SPECIFIED IN PROTOCOL 16

1. INTRODUCTION

This statistical analysis plan (SAP) is developed for the purpose of final analysis and reporting of the study. The SAP provides a detailed, technical elaboration of the statistical analyses of efficacy and safety data as described in the study protocol Version 6.0 dated 14-Oct-2020.

Data being collected up to 31-Aug-2022 will be analyzed per the statistical analysis plan.

2. STUDY OBJECTIVES

2.1. Primary Objectives

- To assess the efficacy of neoadjuvant camrelizumab plus chemotherapy compared with chemotherapy alone, as measured by pathological complete response (pCR), which defined as an absence of viable tumor cells in the surgical specimens from the primary tumor and all sampled regional lymph nodes.

2.2. Secondary Objectives

- To assess the major pathologic response (MPR) in subjects treated with neoadjuvant camrelizumab plus chemotherapy compared with chemotherapy alone.
- To assess the objective response rate (ORR) in subjects treated with neoadjuvant camrelizumab plus chemotherapy compared with chemotherapy alone.
- To assess the event-free survival (EFS) in subjects treated with neoadjuvant camrelizumab plus chemotherapy compared with chemotherapy alone.
- To assess the safety and tolerability of neoadjuvant camrelizumab plus chemotherapy compared with chemotherapy alone.

2.3. Exploratory Objectives

- To assess the surgery related endpoints in subjects treated with neoadjuvant camrelizumab plus chemotherapy compared with chemotherapy alone.

3. STUDY DESIGN AND METHODS

3.1. General Study Design and Plan

This is a randomized, open-label, phase II clinical trial to evaluate the efficacy and safety of neoadjuvant camrelizumab plus chemotherapy in treating patients with resectable stage IIIA/IIIB NSCLC.

Eligible subjects will be randomized at a ratio of 1:1 to receive neoadjuvant camrelizumab plus chemotherapy or chemotherapy alone.

- Subjects assigned to camrelizumab plus chemotherapy group will receive camrelizumab (200 mg) intravenously on day 1 of each 3-week cycle, and nab-paclitaxel (130 mg/m^2 intravenously on days 1 and 8) and platinum (cisplatin: 75 mg/m^2, carboplatin: area under the curve [AUC] 5, or nedaplatin: 100 mg/m^2 intravenously on day 1) every 3 weeks for 3 cycles.
- Subjects assigned to chemotherapy alone group will receive nab-paclitaxel (130 mg/m^2 intravenously on days 1 and 8) and platinum (cisplatin: 75 mg/m^2, carboplatin: area under the curve [AUC] 5, or nedaplatin: 100 mg/m^2 intravenously on day 1) every 3 weeks for 3 cycles.

The choice of platinum will be determined by the investigator. Surgery is planned 3-4 weeks after the completion of neoadjuvant treatment. Tumor responses will be evaluated after the first two cycles of treatment and within 7 days before surgery using radiographic examination according to RECIST version 1.1. A postoperative visit will be performed at 3-6 weeks after surgery.

3.2. Randomization

The eligible subjects will be randomly assigned to the camrelizumab plus chemotherapy group or the chemotherapy alone group at a ratio of 1:1 by permuted block randomization method. The treatment allocation will be implemented via opaque, sealed envelopes.

3.3. Blinding

As this is an open-label study, patients and investigators will not be masked to the treatment allocation.

4. STUDY ENDPOINTS

4.1. Efficacy Endpoint(s)

4.1.1. Primary Efficacy Endpoint(s)

- Pathological complete response (pCR) rate: defined as number of subject with an absence of viable tumor cells in the surgical specimens from the primary tumor and all sampled regional lymph nodes divided by number of subjects who received at least one dose of study treatment for each treatment group.

4.1.2. Secondary Efficacy Endpoint(s)

- Major pathological response (MPR) rate: defined as number of subject with presence of 10% or fewer viable tumor cells in the resected primary tumor specimen and all sampled regional lymph nodes divided by number of subjects who received at least one dose of study treatment for each treatment group.
- Event-free survival (EFS): defined as the time from randomization to any progression of disease before surgery, progression or recurrence of disease after surgery, or death from any cause. Subjects who do not experience progression or recurrence will be censored at the last tumor assessment.
- Objective response rate (ORR): defined as the proportion of subjects whose tumor response (BOR) is CR or PR within 7 days prior to surgery, evaluated by investigator per RECIST 1.1.

4.1.3. Exploratory Efficacy Endpoint(s)

- Surgery related endpoints: include duration from final study treatment to surgery, duration of surgery, length of hospital stay, surgical approach, type of surgery, and proportion of R0 resection.

4.2. Safety Endpoint(s)

Safety endpoints include treatment emergent adverse events (TEAE), treatment related adverse events (TRAE), serious adverse events (SAE), adverse events leading to treatment discontinuation, interruption or dose reduction, immune-mediated adverse events, and death.

In addition, surgery related adverse events are also included.

5. SAMPLE SIZE DETERMINATION

Sample size will be based on the primary endpoint in the primary analysis. pCR rate in the camrelizumab plus chemotherapy group is assumed to be 50% and 18% in the chemotherapy alone group, which can be translated to an odds ration of 4.55. Under these assumption, 80 subjects will provide at least 80% power to detect the difference between two groups with a two-sided alpha level of 0.05 in the primary analysis (i.e., in the full analysis set which is a modified ITT population to include all randomized subjects who received at least 1 dose of study treatment.). Accounting for a potential 15% dropout after randomization, we plan to enroll 94 patients.

6. GENERAL AND STATISTICAL CONSIDERATIONS

6.1. Analysis Sets

6.1.1. Full Analysis Set

Full Analysis Set (FAS) is a modified ITT population to include all randomized subjects who received at least 1 dose of study treatment. FAS will serve as the main analysis set of efficacy analysis.

6.1.2. Surgical Evaluable Set

Surgical Evaluable Set (SES) will include all randomized subjects who received at least 1 dose of study treatment and received surgery.

6.1.3. Safety Analysis Set

The Safety Analysis Set (SS) will include all subjects who received at least 1 dose of the study treatment.

6.2. Statistical Considerations and Reporting Conventions

6.2.1. Statistical Software

All the statistical analysis will be performed with SAS® Version 9.4.

6.2.2. Statistical Summary Conventions

Descriptive statistics including number of subjects with non-missing values, mean, standard deviation, median, minimum, and maximum, will be presented for continuous variables. For categorical variables, frequencies with percentages will be presented. Time-to-events variables will be analyzed by using Kaplan-Meier method and Kaplan-Meier plots will be presented.

6.2.3. General Reporting Conventions

The means and medians should have 1 more decimal place than the original observed values. The standard deviations should have 2 additional decimal place than the original observed values.

Min and Max should have the same decimal place as the original observed values.

p-values should be reported to 3 decimal places. If the p-values be less than 0.001, it should be reported as “<0.001”. While the p-values greater than “0.999” should be reported as “>0.999”.

6.2.4. Subgroups

Subgroup analysis will be conducted for pCR and MPR, which will be described in corresponding sections. It should be noted that the study is not designed to detect the difference in treatment within subgroups. Therefore p-values will not be reported since there is no power calculation and multiplicity adjustment is not considered.

The following subgroups will be assessed for pCR and MPR:

- Age as categorical variable (<65-year, ⩾65-year)
- Sex (Male, Female)
- Disease stage at baseline (IIIA, IIIB)
- Tumor Historic Type (Squamous cell carcinoma, Adenocarcinoma)
- Smoking Status (Current or former smoker, Never smoked)
- PD-L1 expression level (<1%, ⩾1%)

7. STATISTICAL ANALYSIS

7.1. Demographic and Baseline Characteristics

Demographic and baseline characteristics will be summarized by treatment groups in FAS.

- Age as continuous variable (<65-year, ≥65-year)
- Sex (Male, Female)
- Disease stage at baseline (IIIA, IIIB)
- Tumor Historic Type (Squamous cell carcinoma, Adenocarcinoma, Not specified or undifferentiated)
- Smoking Status (Current or former smoker, Never smoked)
- ECOG performance status (0, 1)
- Tumor, Node, Metastasis staging classification (T1N2M0, T2N2M0, T3N1M0, T4N0M0, T4N1M0, T3N2M0)
- PD-L1 expression level (<1%, ≥1%，Unknown)

7.2. Efficacy Analyses

7.2.1. Analysis of Primary Efficacy Endpoints

FAS will be used as the primary analysis set for the primary efficacy analysis.

The primary endpoint is pCR. pCR is defined as number of subjects with an absence of viable tumor cells in the surgical specimens from the primary tumor and all sampled regional lymph nodes divided by number of subjects who received at least one dose of study treatment.

The null hypothesis H_0: $pCR_{treatment} = pCR_{control}$ will be tested using a 2-sided, 5% alpha level, against the alternative hypothesis H_a: $pCR_{treatment} \neq pCR_{control}$.

The null hypothesis is to be rejected at a two-sided significance level of 0.05.

7.2.1.1. Main Analysis of the Primary Efficacy Endpoint

The number and proportion of subjects with pCR in each treatment group will be presented.

The 95% confidence interval of pCR will be estimated by using Clopper-Pearson method. A two sided 95% confidence interval for odds ratio of pCR between the treatment groups will also be computed.

Fisher's exact test will be used to test the difference of pCR between treatment groups.

7.2.1.2. Subgroup Analysis

Subgroup analysis will be performed for the baseline factors listed in section 6.2.4. The unweighted pCR rate differences for camrelizumab plus chemotherapy group versus

chemotherapy alone group and associated 95% confidence interval will be estimated using Miettinen-Nurminen method in each level of the subgroups.

7.2.2. Analysis of Secondary Efficacy Endpoints

7.2.2.1. Major Pathological Response

The primary analysis of major pathological response (MPR) is based on FAS. MPR is defined as number of subjects with presence of 10% or fewer viable tumor cells in the resected primary tumor specimen and all sampled regional lymph nodes divided by number of subjects who received at least one dose of study treatment.

The number and proportion of subjects with MPR in each treatment group will be presented.

The 95% confidence interval of MPR will be estimated by using Clopper-Pearson method. A two sided 95% confidence interval for odds ratio of MPR between the treatment groups will also be computed.

Subgroup analysis for MPR will be performed for the baseline factors listed in section 6.2.4. The unweighted MPR rate differences for camrelizumab plus chemotherapy group versus chemotherapy alone group and associated 95% confidence interval will be estimated using Miettinen-Nurminen method in each level of the subgroups.

7.2.2.2. Event-free Survival

The primary analysis of event-free survival (EFS) is based on FAS. EFS is defined as the time from randomization to any progression of disease before surgery, progression or recurrence of disease after surgery, or death from any cause.

Progression/recurrence of disease will be based on investigators assessment per RECIST 1.1.

The censor rule of EFS is presented as below:

Case number	Description	Censored/ event	Date of censor/event
1	No on-study tumor assessment, and no death	Censored	Randomization date
2	No on-study tumor assessment, and death	Event	Death date
3	With adequate on-study tumor assessment, and recurrence/progression of disease or death	Event	Min(recurrence/progression date, death date)
4	2 or more consecutive missing of tumor assessment before recurrence/progression of disease or death	Censored	Last adequate tumor assessment date before consecutive missing of tumor assessment.

5	With on-study tumor assessment, and no recurrence/progression of disease or death occurred	Censored	Last tumor assessment date

The Kaplan-Meier method will be used to estimate the distribution of EFS in each treatment group, the 95% confidence intervals of median EFS will be calculated by using Brookmeyer and Crowley method, and the 95% confidence intervals of EFS rates by complementary log-log method. The corresponding Kaplan-Meier plots will also be presented. In addition, the unstratified Cox proportional hazards regression model will be used to estimate the hazard ratio and the associated 95% confidence interval.

7.2.2.3. Objective Response Rate (ORR)

Objective response rate (ORR) will be analyzed primarily based on FAS. ORR is defined as the proportion of subjects whose tumor response is CR or PR within 7 days prior to surgery, evaluated by investigator per RECIST 1.1.

The number and proportion of subjects with tumor response of CR, PR, SD, PD and NE in each treatment group will be presented.

The 95% confidence interval of ORR will be estimated by using Clopper-Pearson method.

A two sided 95% confidence interval for odds ratio of ORR between the treatment groups will also be computed.

7.2.3. Analysis of Exploratory Efficacy Endpoints

7.2.3.1. Surgical Related Endpoints

Surgery related endpoints include duration from final study treatment to surgery, duration of surgery, length of hospital stay, surgical approach, type of surgery, and proportion of R0 resection.
Surgery related endpoints will be summarized by the treatment groups descriptively.

7.3. Safety Analyses

All the safety analysis will be performed for SS except for surgery-related adverse events will be analyzed in SES.

Safety endpoints include treatment emergent adverse events (TEAE), treatment related adverse events (TRAE), serious adverse events (SAE), adverse events leading to treatment discontinuation, interruption or dose reduction, immune-mediated adverse events, and death.

In addition, surgery related adverse events are also included.

7.3.1. Adverse Events

AE will be summarized by treatment groups. An AE will be considered as a TEAE occurred or becomes worse in severity after the initiation of study treatment and within 90 days after last study treatment administration.

If the CTCAE grade of an AE is missing, it will be considered as grade 3 AE.

The TEAEs which being recorded as being “related with”, “highly-possibly related with” and “possibly related with” study treatment will be considered as treatment related TEAE (TRAE). If the relationship to the study treatment is missing, it will be considered to be related to study treatment.

A high level summary of the number of subjects with TEAEs will be presented by the treatment groups, including the number and percentage of subjects with:

- Any TEAEs
- TEAEs with CTCAE grade ≥ 3
- TEAE leading to dose reduction
- TEAE leading to treatment interruption
- TEAE leading to treatment discontinuation
- TEAE leading to death
- Serious TEAE (TESAE)
- Immune-mediated TEAE
- Treatment related TEAEs (TRAEs)
- TRAEs with CTCAE grade ≥ 3
- TRAEs leading to dose reduction
- TRAEs leading to treatment interruption
- TRAEs leading to treatment interruption
- TRAEs leading to death
- Serious TRAEs
- Immune-mediated TRAEs

7.3.2. Surgery-related Adverse Events

Surgery-related adverse events will be analyzed in SES.

Number and percentage of subjects with surgery related adverse events for any grade and grade ⩾3 will be summarized by the treatment groups.

8. SUMMARY OF CHANGES TO THE STATISTICAL ANALYSES SPECIFIED IN PROTOCOL

Not applicable.
