# Supplementary material for: Neoadjuvant Camrelizumab Plus Platinum-Based Chemotherapy vs Chemotherapy Alone for Chinese Patients With Resectable Stage IIIA or IIIB (T3N2) Non–Small Cell Lung Cancer: The TD-FOREKNOW Randomized Clinical Trial
Source: JAMA Oncol. 2023 Aug 3;9(10):1348–55. doi: 10.1001/jamaoncol.2023.2751 (PMC10401395; doi:10.1001/jamaoncol.2023.2751)
Supplement: Supplement 2. — eAppendix. Supplemental Methods eTable. Surgical Outcomes eFigure 1. Waterfall Plot of Camrelizumab Plus Chemotherapy (A) and Chemotherapy Alone (B) eFigure 2. Forest Plot of Subgroup Analysis for Pathological Complete Response Rate eFigure 3. Forest Plot of Subgroup Analysis for Major Pathological Response Rate [file jamaoncol-e232751-s002.pdf]

Supplementary Online Content

Lei J, Zhao J, Gong L, et al. Neoadjuvant camrelizumab plus platinum-based chemotherapy vs chemotherapy alone for Chinese patients with resectable stage IIIA or IIIB (T3N2) non–small cell lung cancer: the TD-FOREKNOW randomized clinical trial. *JAMA Oncol.* Published online August 3, 2023. doi:10.1001/jamaoncol.2023.2751

eAppendix. Supplemental Methods
eTable. Surgical Outcomes
eFigure 1. Waterfall Plot of Camrelizumab Plus Chemotherapy (A) and Chemotherapy Alone (B)
eFigure 2. Forest Plot of Subgroup Analysis for Pathological Complete Response Rate
eFigure 3. Forest Plot of Subgroup Analysis for Major Pathological Response Rate

This supplementary material has been provided by the authors to give readers additional information about their work.

eAppendix. Supplemental Methods

Dose modifications

Dose delay or interruption of camrelizumab was allowed in the event of an adverse event, but dose reductions were not permitted. For nab-paclitaxel, two dose reductions were allowed (to 100 mg/m^2 and 65 mg/m^2) depending on tolerance. Dose adjustment of platinum was determined by the investigators according to medicine instruction and clinical practice.

Assessments

Tumor response was evaluated within 7 days before surgery using radiographic examination according to RECIST version 1.1. A postoperative visit was performed at 3-6 weeks after surgery. Pathological response was assessed by local pathologists who measured the percentage of residual viable tumor in resected primary tumors and sampled regional lymph nodes, followed by confirmation from two senior pathologists. Abnormalities in laboratory parameters, electrocardiogram and vital signs were monitored before each treatment cycle, within 7 days before surgery, and 3-6 weeks after surgery. Surgical complications and mortality were monitored during the first 30 days after surgery, which were graded as per the Clavien-Dindo classification. Adverse events (AEs) were recorded until 90 days after the last dose of study treatment and graded according to the National Cancer Institute Common Terminology Criteria for Adverse Events (NCI CTCAE) version 5.0. The PD-L1 tumor cell proportion score (TPS) was assessed using the VENTANA PD-L1 (SP263) Assay (Ventana Medical Systems, Inc. Tucson, AZ, USA).

© 2023 Lei J et al. *JAMA Oncology.*

eTable. Surgical Outcomes

	Camrelizumab Plus Chemotherapy (*n* = 40)	Chemotherapy (*n* = 42)
Duration from final treatment to surgery, weeks		
median (IQR)	4.7 (4.0-5.6)	4.6 (4.0-4.9)
< 4	6 (15.0)	6 (14.3)
4-6	29 (72.5)	35 (83.3)
> 6	5 (12.5)	1 (2.4)
Type of surgery, *n* (%)		
Video-assisted thoracoscopic surgery	22 (55.0)	26 (61.9)
Thoracotomy	15 (37.5)	14 (33.3)
Video-assisted thoracoscopic surgery to Thoracotomy	3 (7.5)	2 (4.8)
Surgical resection, *n* (%)		
Lobectomy	36 (90.0)	34 (81.0)
Pneumonectomy	4 (10.0)	8 (19.0)
Operative time (min), median (IQR)	151.5 (120.0-198.8)	150.0 (130.0-196.3)
R0 resection, *n* (%)	37 (92.5)	36 (85.7)
Downstaging, *n* (%)	23 (53.5)	20 (44.4)
Length of hospital stay after surgery (days), median (IQR)	10 (8-12)	9 (7-11)

IQR, interquartile range.

© 2023 Lei J et al. *JAMA Oncology.*

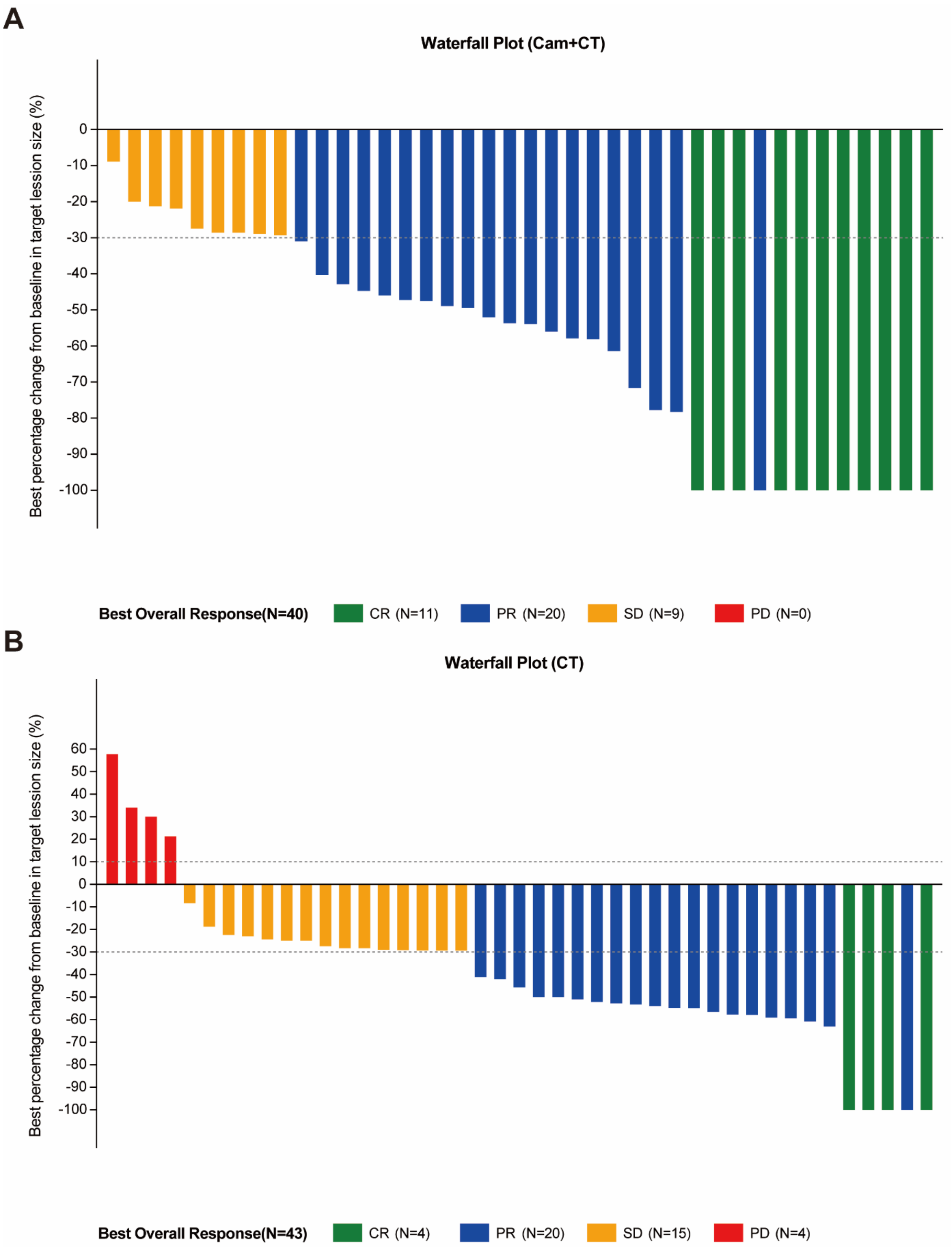

eFigure 1. Waterfall Plot of Camrelizumab Plus Chemotherapy (A) and Chemotherapy Alone (B)

Cam+CT, camrelizumab plus chemotherapy; CR, complete response; CT, chemotherapy; PD, progressive disease; PR, partial response; SD, stable disease.

© 2023 Lei J et al. *JAMA Oncology.*

Subgroup	No.of Patients	Pathologic Complete Response Rate,% (95% CI) Cam+chemo (n=43)	Chemo (n=45)	Pathologic Complete Response Rate Difference,% (95% CI)	Odds Ratio (95% CI)
**Overall**	88	32.6 (19.1-48.5)	8.9 (2.5-21.2)	23.7 (7.1-40.2)	4.95 (1.35-22.37)
**Age**					
<65 years	63	34.4 (18.6-53.2)	12.9 (3.6-29.8)	21.5 (0.3-41.5)	3.5 (0.9-17.1)
≥65 years	25	27.3 (6.0-61.0)	0.0 (0.0-23.2)	27.3 (1.7-57.1)	NE (0.8-NE)
**Sex**					
Male	74	35.3 (19.7-53.5)	10.0 (2.8-23.7)	25.3 (6.6-43.9)	4.9 (1.3-23.0)
Female	14	22.2 (2.8-60.0)	0.0 (0.0-52.2)	22.2 (-27.6-55.9)	NE (0.2-NE)
**Disease stage at baseline**					
IIIA	66	30.0 (14.7-49.4)	8.3 (1.8-22.5)	21.7 (3.0-41.1)	4.7 (1.0-29.5)
IIIB	22	38.5 (13.9-68.4)	11.1 (0.3-48.2)	27.4 (-12.8-57.7)	5.0 (0.4-265.5)
**Tumor Historic Type**					
Squamous cell carcinoma	59	37.0 (19.4-57.6)	12.5 (3.5-29.0)	24.5 (2.6-45.8)	4.1 (1.0-20.4)
Adenocarcinoma	26	20.0 (4.3-48.1)	0.0 (0.0-28.5)	20.0 (-9.0-45.7)	NE (0.4-NE)
**Smoking Status**					
Current or former smoker	68	35.5 (19.2-54.6)	10.8 (3.0-25.4)	24.7 (4.9-44.3)	4.5 (1.1-21.7)
Never smoked	20	25.0 (5.5-57.2)	0.0 (0.0-36.9)	25.0 (-12.2-53.9)	NE (0.4-NE)
**PD-L1 expression level**					
<1%	15	57.1 (18.4-90.1)	12.5 (0.3-52.7)	44.6 (-5.0-77.6)	9.3 (0.5-523.5)
≥1%	27	56.3 (29.9-80.2)	18.2 (2.3-51.8)	38.1 (-0.4-65.6)	5.8 (0.8-67.4)

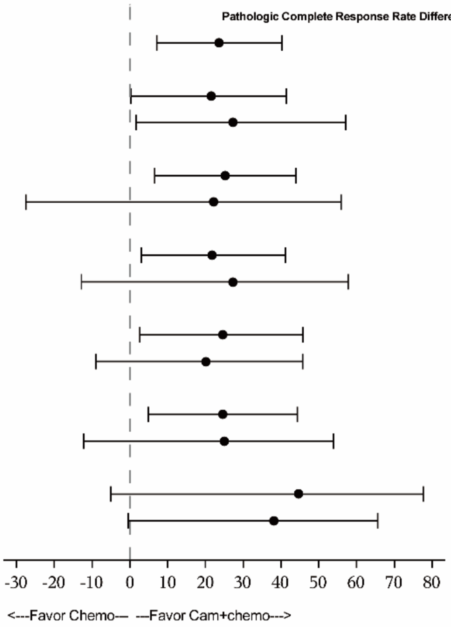

eFigure 2. Forest Plot of Subgroup Analysis for Pathological Complete Response Rate
Cam+chemo, camrelizumab plus chemotherapy; Chemo, chemotherapy; CI, confidence interval; NE, not evaluable; PD-L1, programmed cell death ligand 1.

© 2023 Lei J et al. *JAMA Oncology.*

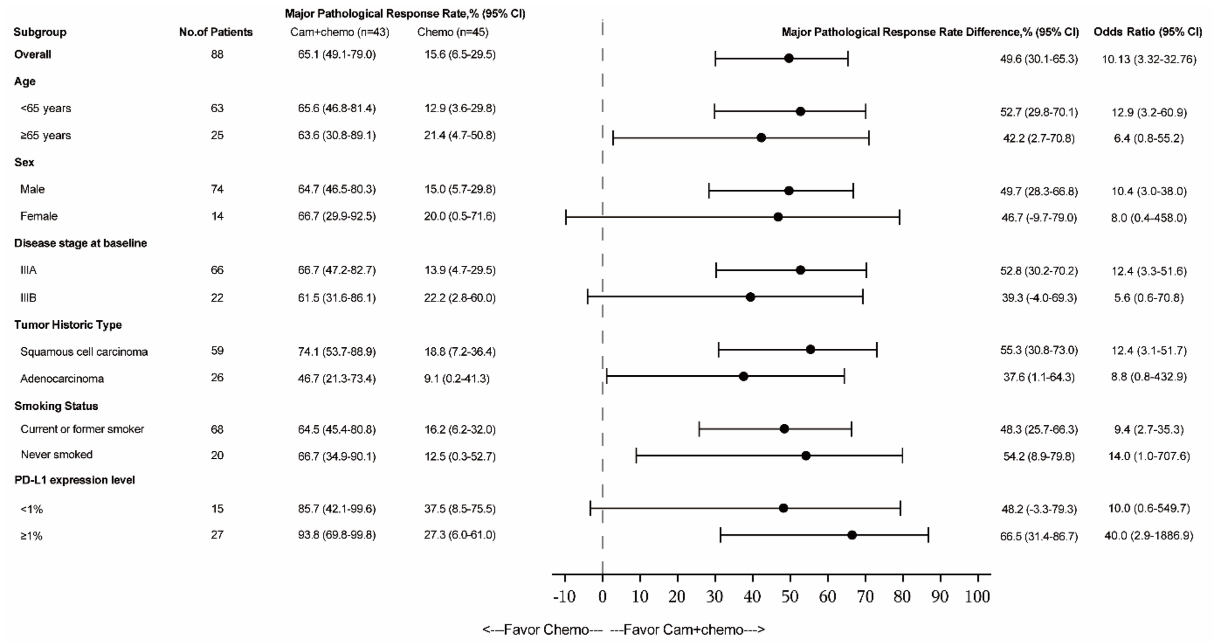

eFigure 3. Forest Plot of Subgroup Analysis for Major Pathological Response Rate

Cam+chemo, camrelizumab plus chemotherapy; Chemo, chemotherapy; CI, confidence interval; PD-L1, programmed cell death ligand 1.

© 2023 Lei J et al. *JAMA Oncology.*
