# Supplementary material for: Neoadjuvant Camrelizumab Plus Platinum-Based Chemotherapy vs Chemotherapy Alone for Chinese Patients With Resectable Stage IIIA or IIIB (T3N2) Non–Small Cell Lung Cancer: The TD-FOREKNOW Randomized Clinical Trial
Source: JAMA Oncol. 2023 Aug 3;9(10):1348–55. doi: 10.1001/jamaoncol.2023.2751 (PMC10401395; doi:10.1001/jamaoncol.2023.2751)
Supplement: Supplement 3. — Data Sharing Statement [file jamaoncol-e232751-s003.pdf]

Data Sharing Statement

Lei. Neoadjuvant Camrelizumab Plus Platinum-Based Chemotherapy vs Chemotherapy Alone for Chinese Patients With Resectable Stage IIIA or IIIB (T3N2) Non–Small Cell Lung Cancer. *JAMA Oncol*. Published August 03, 2023. doi:10.1001/jamaoncol.2023.2751

Data
Data available: No
